# Juvenile murine models of prediabetes and type 2 diabetes develop neuropathy

**DOI:** 10.1242/dmm.037374

**Published:** 2018-12-18

**Authors:** Phillipe D. O'Brien, Lucy M. Hinder, Amy E. Rumora, John M. Hayes, Jacqueline R. Dauch, Carey Backus, Faye E. Mendelson, Eva L. Feldman

**Affiliations:** Department of Neurology, University of Michigan, Ann Arbor, MI 48109-2200, USA

**Keywords:** Mouse models, Obesity, Peripheral neuropathy, Prediabetes, Type 2 diabetes

## Abstract

Peripheral neuropathy (neuropathy) is a common complication of obesity and type 2 diabetes in children and adolescents. To model this complication in mice, 5-week-old male C57BL/6J mice were fed a high-fat diet to induce diet-induced obesity (DIO), a model of prediabetes, and a cohort of these animals was injected with low-dose streptozotocin (STZ) at 12 weeks of age to induce hyperglycemia and type 2 diabetes. Neuropathy assessments at 16, 24 and 36 weeks demonstrated that DIO and DIO-STZ mice displayed decreased motor and sensory nerve conduction velocities as early as 16 weeks, hypoalgesia by 24 weeks and cutaneous nerve fiber loss by 36 weeks, relative to control mice fed a standard diet. Interestingly, neuropathy severity was similar in DIO and DIO-STZ mice at all time points despite significantly higher fasting glucose levels in the DIO-STZ mice. These mouse models provide critical tools to better understand the underlying pathogenesis of prediabetic and diabetic neuropathy from youth to adulthood, and support the idea that hyperglycemia alone does not drive early neuropathy.

This article has an associated First Person interview with the first author of the paper.

## Abstract (precis)

**Summary:** The mouse models described in this paper provide critical tools to better understand the underlying pathogenesis of prediabetic and diabetic neuropathy from youth to adulthood.

## INTRODUCTION

According to a recent study released by the Centers for Disease Control and Prevention (CDC) as part of the Youth Risk Behavior Surveillance System, 15.6% of adolescents in the US are overweight and an additional 14.8% are obese (www.cdc.gov). Among children and adolescents ranging in age from 5 to 18 years old, obesogenic behaviors (including sedentary behavior, poor diet and physical inactivity) (Avery et al., 2017; Leech et al., 2014, 2015; Santaliestra-Pasías et al., 2015) underlie the increasing prevalence of prediabetes and type 2 diabetes in adolescents, especially among certain racial and ethnic groups (Kao and Sabin, 2016; Marley and Metzger, 2015; Tieh and Dreimane, 2014; Pulgaron and Delamater, 2014; Mayer-Davis et al., 2017). Of the 27,000 participants in The SEARCH for Diabetes in Youth study (SEARCH), the incidence of type 2 diabetes is as high as 8.9% in youth between 10 and 19 years of age. Among those with type 2 diabetes, the most prevalent complication is neuropathy, present in over 22% of the study population (Jaiswal et al., 2017; Jensen and Dabelea, 2018).

Neuropathy is defined as the loss of peripheral nerve function in the extremities in a distal to proximal pattern that is characterized predominantly by sensory symptoms. There is a high degree of morbidity associated with the condition, including numbness, pain, poor balance and co-ordination, frequent falls, and ulceration in the lower extremities over time that can lead to amputation (Callaghan et al., 2012a). Despite the high prevalence of neuropathy in patients with obesity and diabetes, its exact etiology remains unknown, with no effective therapeutic options available for either children or adults other than lifestyle intervention (Diabetes Prevention Program Research Group, 2015; The Look AHEAD Research Group, 2017; Smith et al., 2006).

In response to the global burden of diabetes and its complications, including neuropathy, the National Institutes of Health formed The Diabetic Complications Consortium (www.diacomp.org) in 2000 to develop novel animal models of diabetic complications in order to understand disease pathogenesis so that effective therapies can be developed and translated to patients (O'Brien et al., 2014b). For neuropathy, these models should manifest features similar to those found in disease, including sensory deficits, decreased nerve conduction velocities and anatomical evidence of nerve fiber loss (Biessels et al., 2014). Among those most extensively used in research, the *db/db* and *ob/ob* leptin-based models of type 2 diabetes successfully simulate rapid-onset, severe type 2 diabetes and neuropathy, while the high-fat-diet-fed mouse model of diet-induced obesity (DIO) simulates obesity-driven prediabetes and neuropathy (Sullivan et al., 2007; O'Brien et al., 2014a; Hinder et al., 2017). Although extremely important research has been performed using these models, none follow the natural progression from obesity and impaired glucose tolerance to type 2 diabetes. Thus, to more closely model disease progression, investigators have recently incorporated the use of streptozotocin (STZ) treatment in non-genetic animal models of obesity to bring about a greater degree of hyperglycemia in DIO mice. The protocol, originally developed by Luo and colleagues (Luo et al., 1998) and later refined by Gilbert et al. (2011), involves administering a low dose of STZ to DIO mice, who at baseline exhibit obesity and impaired glucose tolerance. This results in impairment of insulin secretion and hyperglycemia, similar to what is seen at the later stages of type 2 diabetes. Several groups have now reported using the DIO-STZ model in adult mice and rats to explore disease pathogenesis, primarily emphasizing metabolic control (Lin et al., 2015; Nath et al., 2017; Li et al., 2014; Wang et al., 2014; Bansal et al., 2012; Yu et al., 2017), and, although its use in diabetic complications is more limited (Guilford et al., 2011; Davidson et al., 2018; Shevalye et al., 2015), Barrière et al*.* recently demonstrated that DIO-STZ rats develop complications of type 2 diabetes, including diabetic nephropathy, retinopathy and neuropathy (Barrière et al., 2018).

Given that our reports from SEARCH in children and adolescents (Jaiswal et al., 2013; Jaiswal et al., 2017) emphasize a critical need to understand neuropathy onset and progression in youth, the goal of the current study was to develop a mouse model that reflects the development of neuropathy from youth to adulthood in both prediabetes and diabetes. We therefore modified an established DIO-STZ mouse model of adult-onset type 2 diabetes and neuropathy (Yorek et al., 2015) and generated type 2 diabetes in adolescent mice to examine neuropathy progression from prediabetes to type 2 diabetes. This first-in-kind model will provide a new tool for understanding neuropathy in youth with prediabetes and type 2 diabetes, and provide a framework for much-needed therapeutic development.

## RESULTS

### Metabolic phenotyping

**Fig. 1.**
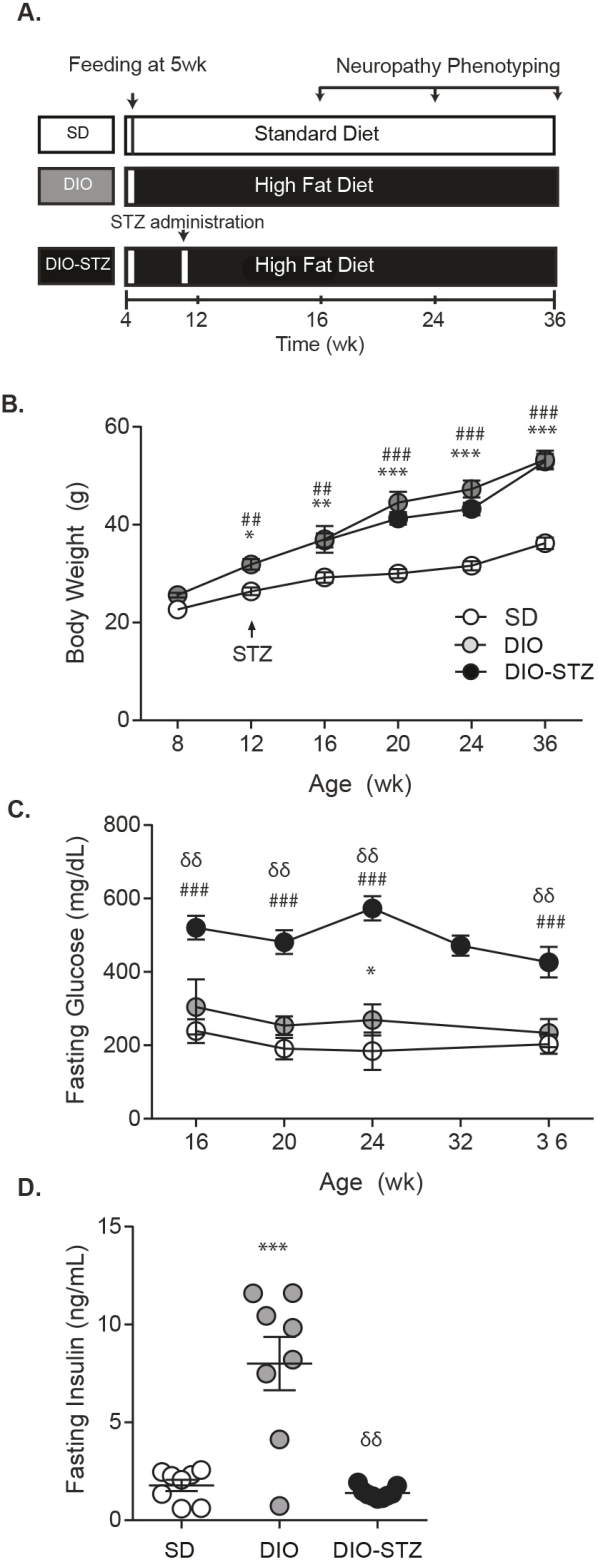
**Metabolic phenotyping.** (A) Experimental design. (B) Body weight and (C) glucose were examined longitudinally in DIO-STZ mice, DIO mice and mice fed a standard diet (SD). (D) At 36 weeks, plasma insulin was measured. Data are mean±s.e.m. One-way ANOVA with Tukey's multiple comparisons test, *P<0.05, **P<0.01 or ***P<0.001 vs SD; ^##^*P*<0.01 or ^###^*P*<0.001 DIO-STZ vs SD; ^δδδ^*P*<0.001 DIO-STZ vs DIO. Sample sizes: (B) 8, 12, 20, 24 weeks: SD *n*=10, DIO *n*=10, DIO-STZ *n*=10; 16 weeks: SD *n*=8, DIO *n*=8, DIO-STZ *n*=8; 36 weeks: SD *n*=8, DIO *n*=10, DIO-STZ *n*=9; (C) 16 weeks: *n*=8/group; 20 and 24 weeks: *n*=10/group; 32 weeks: DIO-STZ *n*=9; 36 weeks: SD *n*=8, DIO *n*=10, DIO-STZ *n*=7; (D) SD *n*=8, DIO *n*=8, DIO-STZ *n*=9.

Our study consisted of three groups of C57BL/6J mice: control mice fed a standard diet (SD mice), mice fed a high-fat (HF) diet to induce DIO (DIO mice), and DIO mice injected with STZ (DIO-STZ mice) to model T2D (Fig. 1A). Mice were fed either a standard or HF diet starting at 5 weeks of age and, at 12 weeks, a subset of DIO mice were injected with STZ to model development of a diabetes-like phenotype during adolescence. While STZ is commonly used to induce a type 1 diabetic phenotype, the low-dose strategy adopted here causes a mild impairment of insulin secretion to bring about hyperglycemia.

During the course of the study, all groups displayed a steady increase in weight (SD, DIO, DIO-STZ; Fig. 1B); however, beginning at 12 weeks, both DIO and DIO-STZ mice showed similar, significant increases in weight relative to SD mice. By 36 weeks, DIO and DIO-STZ mice showed no difference in weight (both were ∼53 g), being ∼17 g heavier than SD mice. Fasting glucose levels of DIO-STZ mice were elevated (>400 mg/dl) at each time point relative to the levels observed in DIO and SD mice, which were in the region of 200-300 mg/dl (Fig. 1C). At 36 weeks, fasting plasma insulin was determined (Fig. 1D) and, as anticipated, DIO mice displayed robust hyperinsulinemia, with a 4.7-fold increase in insulin compared to SD mice. Fasting insulin levels in DIO-STZ mice (1.43±0.1 ng/ml) were not significantly different from controls (1.9±0.3 ng/ml), which was expected due to the STZ mode of action, which limits insulin secretion.

**Fig. 2.**
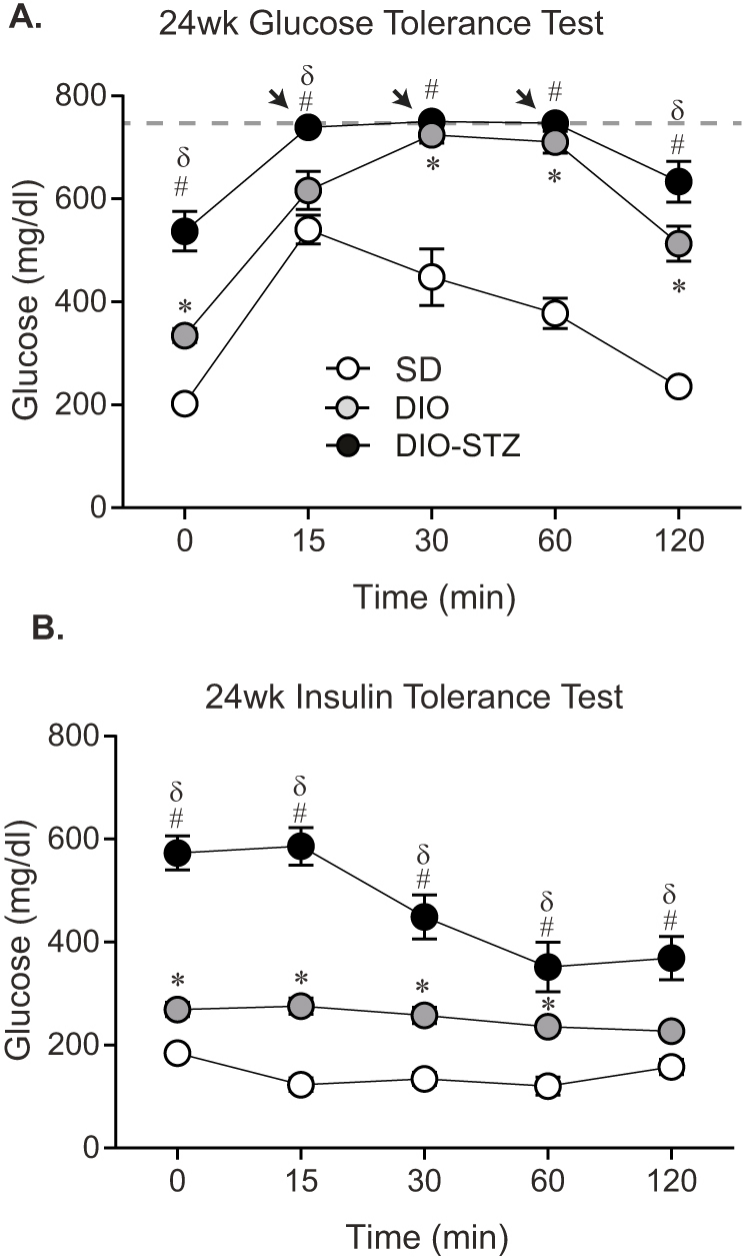
**Assessment of glucose intolerance in DIO and DIO-STZ mice.** GTT (A) and ITT (B) were performed at 24 weeks. Data are mean±s.e.m. One-way ANOVA with Tukey's multiple comparisons test, *P<0.05, DIO vs SD; ^#^*P*<0.05 DIO-STZ vs SD; ^δ^*P*<0.05 DIO-STZ vs DIO. Several DIO-STZ mice injected with glucose had elevated glucose levels that were above the limits of detection (>750 mg/dl; notated by arrows in A); therefore, for the purposes of statistical testing, their values were recorded as 750 mg/dl. Sample sizes: (A) 0 min: *n*=10/group; 15-120 min: SD *n*=10, DIO *n*=8, DIO-STZ *n*=10; (B) 0-15 min: *n*=10/group; 30-60 min: SD *n*=9, DIO *n*=10, DIO-STZ *n*=10; 120 min: SD *n*=8, DIO *n*=10, DIO-STZ *n*=10.

Glucose and insulin tolerance testing (GTT and ITT, respectively) were then performed to assess glucose disposal and insulin resistance, respectively, both of which are hallmarks of T2D (Fig. 2). DIO-STZ mice had impaired glucose tolerance at 24 weeks, similar to DIO mice, with a decrease in glucose levels only observed after the 60-min time point (Fig. 2A). It should be noted that DIO-STZ mice had glucose measurements >750 mg/dl, which is above the limits of detection, thus making it difficult to accurately compare them to DIO mice. The ITT demonstrated that DIO-STZ mice were responsive to an insulin bolus at 24 weeks (Fig. 2B). Between the 15- to 30-min time points, there was a drop in glucose levels from 586±36.4 mg/dl to 449±42.7 mg/dl in DIO-STZ mice. At the same time interval, a drop in glucose levels was not observed in DIO mice, suggesting that DIO-STZ mice are more insulin sensitive compared to DIO mice.

**Fig. 3.**
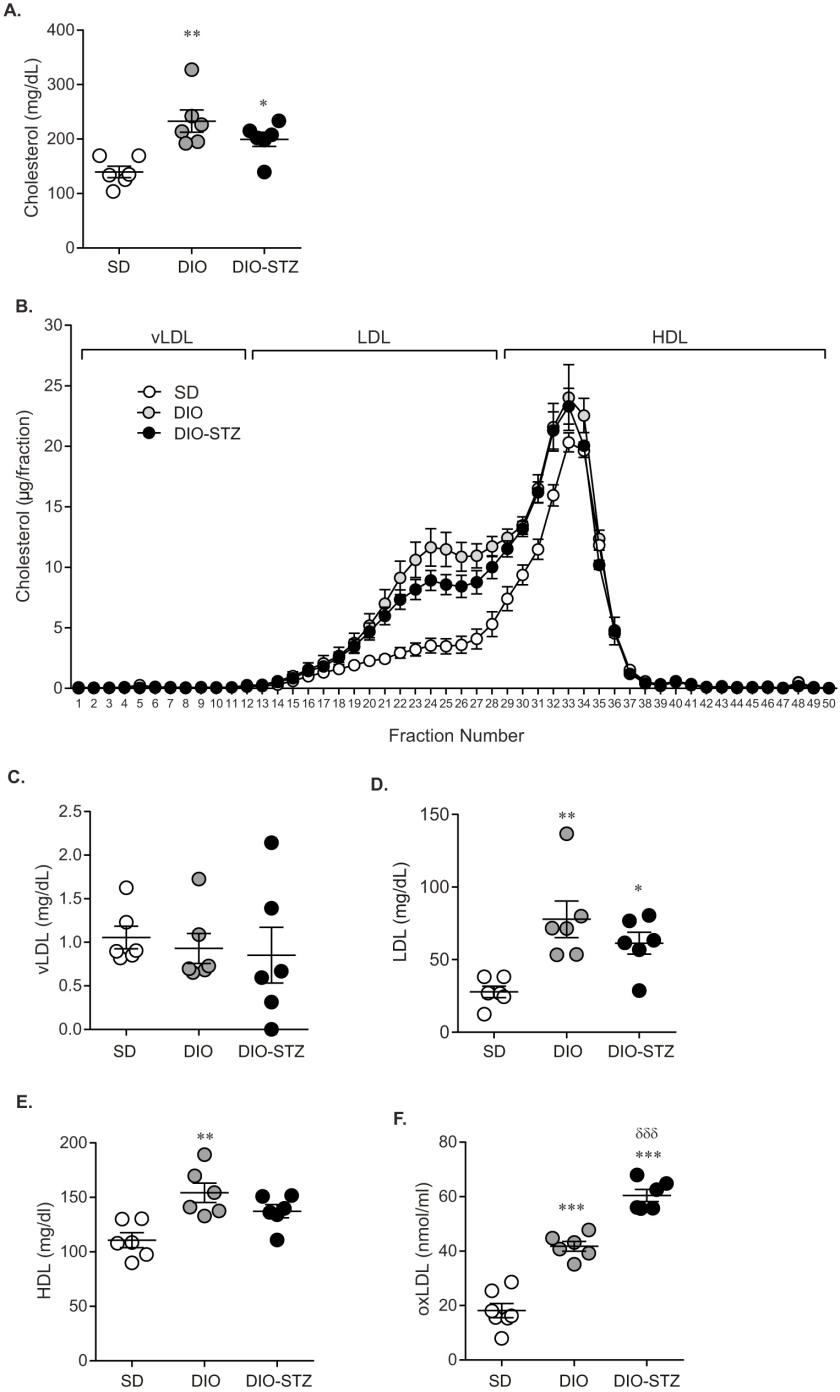
**DIO-STZ C57BL/6J mice exhibit dyslipidemia.** (A) At 36 weeks, total cholesterol was measured and (B) FPLC-cholesterol was performed to derive lipoprotein fractions, including (C) vLDL, (D) LDL and (E) HDL. (F) Plasma oxLDL levels were also measured at 36 weeks. Data are mean±s.e.m. One-way ANOVA with Tukey's multiple comparisons test, *P<0.05, **P<0.01 or ***P<0.001 vs SD; ^δδδ^*P*<0.001 DIO-STZ vs DIO. Sample sizes: (A-E) SD *n*=6, DIO *n*=6, DIO-STZ *n*=6; (F) SD *n*=7, DIO *n*=6, DIO-STZ *n*=6.

As emerging data support dyslipidemia as a major contributor to neuropathy, plasma cholesterol profiling was next assessed. At 36 weeks, total cholesterol was higher in DIO (232.9±20.4 mg/dl) and DIO-STZ mice (199.4±13.0 mg/dl) compared to SD mice (139.6±10.5 mg/dl) (Fig. 3A). DIO and DIO-STZ mice also had similar fast-protein liquid chromatography (FPLC) cholesterol profiles that were significantly different from SD mice (Fig. 3B), with increases in cholesterol in the low-density lipoprotein (LDL) to high-density lipoprotein (HDL) range. Indeed, upon closer examination of the specific very LDL (vLDL), LDL and HDL fractions, LDL and HDL levels were increased in DIO and DIO-STZ mice compared to SD mice (Fig. 3C-E). Specifically, looking at the contributions of cholesterol in the LDL fraction, DIO-STZ and DIO mice had LDL-cholesterol levels of 61.3±7.5 mg/dl and 77.8±12.5 mg/dl, respectively, compared to SD mice, which had a mean of 27.8±3.9 mg/dl. LDL, in the presence of oxidative stress, spontaneously oxidizes to form oxLDL, a lipid factor considered to be particularly injurious to neurons. Plasma oxLDL was significantly elevated in DIO mice compared to SD mice (2.3-fold increase) and even further elevated in DIO-STZ mice (3.3-fold increase) (Fig. 1F). Collectively, these data show that juvenile DIO mice treated with STZ develop metabolic changes similar to those of HF-fed DIO mice, but also develop robust hyperglycemia.

### Peripheral neuropathy

**Fig. 4.**
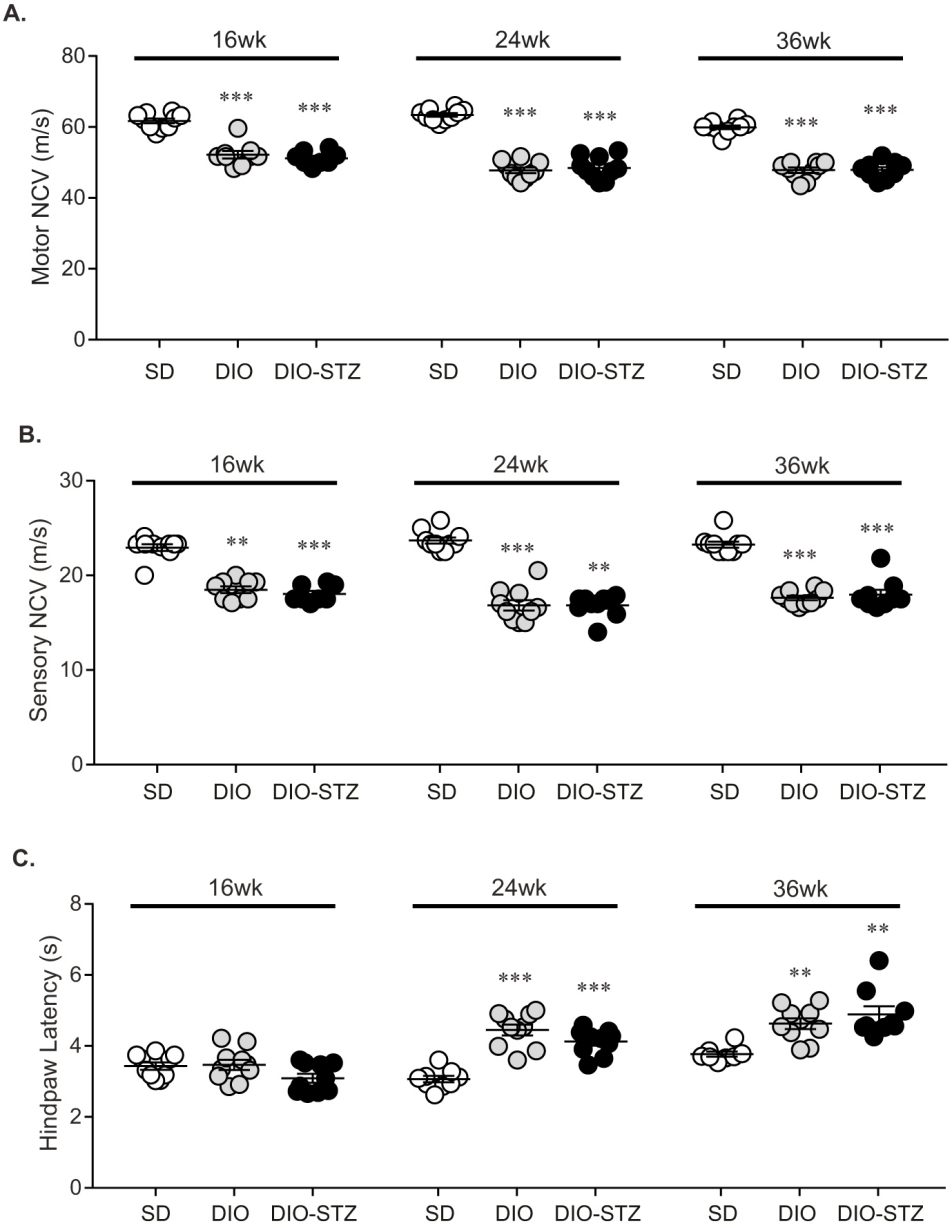
**Neuropathy phenotyping.** Measures of peripheral neuropathy were performed at 16, 24 and 36 weeks: (A) motor and (B) sensory NCV, and (C) hindpaw withdrawal latency. Data are mean±s.e.m. One-way ANOVA with Tukey's multiple comparisons test, **P<0.01 or ***P<0.001 vs SD. Sample sizes: (A) 16 weeks: SD *n*=10, DIO *n*=9, DIO-STZ *n*=8; 24 weeks: SD *n*=10, DIO *n*=10, DIO-STZ *n*=10; 36 weeks: SD *n*=10, DIO *n*=10, DIO-STZ *n*=9; (B) 16 weeks: SD *n*=10, DIO *n*=9, DIO-STZ *n*=10; 24 weeks: SD *n*=10, DIO *n*=10, DIO-STZ *n*=10; 36 weeks: SD *n*=10, DIO *n*=10, DIO-STZ *n*=9; (C) 16 weeks: SD *n*=10, DIO *n*=10, DIO-STZ *n*=10; 24 weeks: SD *n*=9, DIO *n*=10, DIO-STZ *n*=10; 36 weeks: SD *n*=8, DIO *n*=10, DIO-STZ *n*=9.

**Fig. 5.**
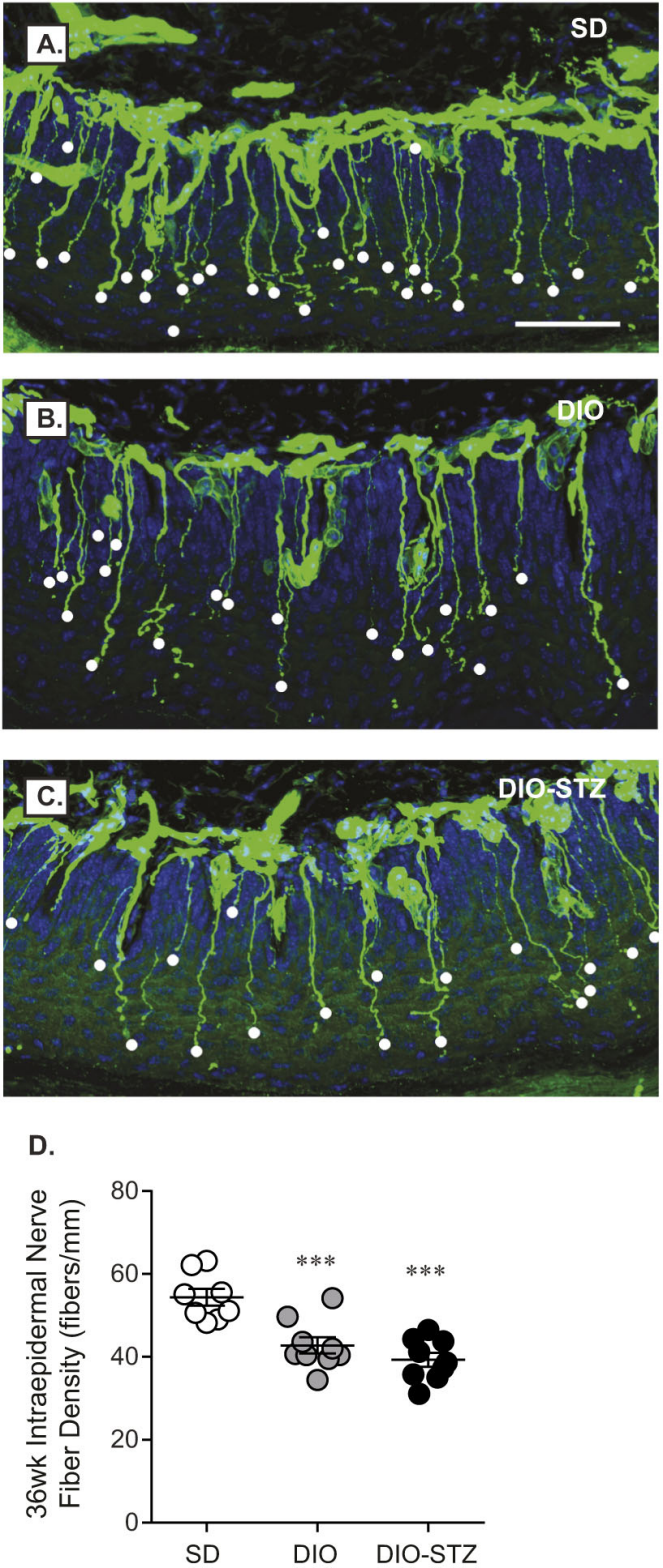
**Intraepidermal nerve fiber loss in DIO and DIO-STZ mice.** Representative images of each group (3 images per mouse) highlighting decreased IENFD in (A) SD, (B) DIO and (C) DIO-STZ mice at 36 weeks. Scale bar: 50 µm. (D) Individual fibers (white dots, A-C) were counted and plotted as described in the Materials and Methods. Data are mean±s.e.m. One-way ANOVA with Tukey's multiple comparisons test, ***P<0.001 vs SD. Sample sizes: (D) SD *n*=8, DIO *n*=9, DIO-STZ *n*=9.

DIO and DIO-STZ mice had motor and sensory nerve conduction velocity (NCV) deficits at 16, 24 and 36 weeks when compared to SD mice (Fig. 4A,B). Interestingly, there was no significant difference in motor or sensory NCVs between both DIO and DIO-STZ mice at any time point, despite each group having profound differences in metabolic measures. Also worth noting is that motor and sensory NCV did not decrease over time in either group. Significant increases in hindpaw withdrawal latency were also evident in older DIO and DIO-STZ mice compared to SD mice (Fig. 4C), highlighting development of thermal hypoalgesia, a measure of sensory loss. Assessment of intraepidermal nerve fiber density (IENFD) in the footpad of the mouse hindpaw provides anatomical evidence of nerve fiber loss. IENFD was significantly lower in DIO (42.8±1.9 fibers/mm) and DIO-STZ (39.3±1.7 fibers/mm) mice compared to SD mice (54.4±2.0 fibers/mm) at 36 weeks (Fig. 5). Similar to NCV and hindpaw measures, there was no discernable difference in IENFD between DIO and DIO-STZ mice. These data indicate that, despite differences in metabolic markers of T2D in DIO mice treated with STZ, adolescent DIO and DIO-STZ mice both develop a similar degree of peripheral neuropathy.

## DISCUSSION

A murine model of neuropathy early in the lifespan of the mouse, equivalent to adolescence in humans, can provide a unique and needed resource for the study of diabetes and its complications. In the current study, we longitudinally phenotyped neuropathy in non-genetic murine models of prediabetes (DIO) and type 2 diabetes (DIO-STZ) over a period spanning adolescence to adulthood. Our data revealed an early onset and similar degree of neuropathy among the two disease cohorts of adolescent mice that persisted to adulthood with similar severity in both models, despite the robust hyperglycemia of mice with the type 2 diabetes phenotype. Thus, these juvenile DIO and DIO-STZ mice uniquely parallel the progression of the human condition and represent a valuable tool to study how prediabetes and type 2 diabetes contribute to the development of neuropathy in youth.

Given that an animal model of disease should recapitulate the human disorder as closely as possible, the Neurodiab Study Group of the European Association for the Study of Diabetes determined that the diagnostic criteria required for a murine model of diabetic neuropathy should include assessments of behavior, motor and sensory NCVs, and nerve structure (Biessels et al., 2014). Neuropathy is defined as the presence of valid and statistically different values in two of the three parameters between the control and diabetic animals. Hindpaw latency and assessment of IENFD are the recommended behavioral and structural assessments, and both were used in this study. We also completed the recommended motor and sensory NCV studies, and completed serial measurements on all animals.

Using these established criteria, we confirmed neuropathy across a spectrum of ages corresponding to the transition between adolescence and adulthood in non-genetic prediabetic and diabetic animals. Animals in our study began a HF diet at 5 weeks of age and, by 12 weeks of age, HF-diet-fed animals weighed significantly more than animals fed normal chow, i.e. these animals had DIO. At 12 weeks, a cohort of DIO mice received STZ. By 16 weeks of age, the murine equivalent of adolescence (Dutta and Sengupta, 2016), all animals were obese and the DIO-STZ animals displayed elevated fasting glucose levels, frank glucose intolerance, and type 2 diabetes. Moreover, both the adolescent 16-week DIO and DIO-STZ animals had neuropathy and displayed sensory and motor NCV deficits. These deficits were similar to those reported by the Yorek laboratory in adult DIO and DIO-STZ mice (Shevalye et al., 2015), where animals began a HF diet at 13 weeks of age and DIO-STZ mice received low-dose STZ at 21 weeks. Having a mouse model that exerts a similar neuropathy phenotype across laboratories demonstrates that the model can easily be recapitulated, especially when investigators source animals of the same strain and from the same supplier, feed the mice the same diet, and use similar STZ paradigms. Small alterations in the protocol used to generate the model, however, may not yield the same phenotype. For example, comparing DIO mice across laboratories, we consistently observed neuropathy at 16 weeks while Wright and colleagues failed to see neuropathy in their respective animals (Guilford et al., 2011). These differences in neuropathy presentation between research groups may be due to one or more of the factors mentioned above, and future studies comparing vendors, timing of HF feeding, and dietary fat content are needed as murine models from youth to adulthood are developed for preclinical therapeutic testing.

Our study is the first to confirm neuropathy in both non-genetic DIO and DIO-STZ adolescent mice. The increasing prevalence of obesity, prediabetes and type 2 diabetes in children and adolescents is an immediate and serious concern, and neuropathy is a common complication in this population (Dabelea et al., 2014; Jaiswal et al., 2013, 2017). These animals will provide a unique resource to investigate not only the pathogenesis of neuropathy but also potential early therapeutic interventions in youth. This is extremely important in light of the recent American Diabetes Association (ADA) position statement on the diagnosis and treatment of diabetic neuropathy. The ADA recommends that all patients with new-onset type 2 diabetes be immediately assessed for neuropathy, along with all prediabetic patients with symptoms (Pop-Busui et al., 2017). With the high prevalence of neuropathy in adolescents with type 2 diabetes, reported as 22% in the SEARCH cohort (Jaiswal et al., 2017; Jensen and Dabelea, 2018), animal models like the one presented in the current study are needed to understand what early interventions could provide therapeutic benefit.

For both prediabetes and diabetes, the ADA recommends lifestyle interventions, with an emphasis on diet and exercise. These recommendations come in light of the fact that glucose control has a modest or no effect on preventing the progression of type 2 diabetic neuropathy in multiple large clinical trials in man (Callaghan et al., 2012b). These human data support our findings where neuropathy severity is not impacted by glycemia in the DIO and DIO-STZ animals. There are no studies to show that glucose control alone can prevent diabetic neuropathy in murine models of type 2 diabetes (O'Brien et al., 2014b). However, glucose control can partially prevent diabetic neuropathy in murine models of type 1 diabetes (Yorek et al., 2014). Interestingly, these same findings occur in man, where glucose control ameliorates diabetic neuropathy in patients with type 1 diabetes (Callaghan et al., 2012b). The concordance of the non-genetic animal models of diabetes and diabetic neuropathy with the findings from large clinical trials in type 1 and type 2 diabetes further supports the utility of using non-genetic murine models.

We next assessed glucose intolerance in all animals as they matured into young (24 weeks) adults (Dutta and Sengupta, 2016), and observed that DIO animals developed prediabetes and DIO-STZ animals had more profound hyperglycemia, with continued features of type 2 diabetes. Indeed, at all ages, DIO-STZ animals had higher glycemia levels than DIO mice, as previously reported (Yorek et al., 2015; Guilford et al., 2011). Despite significant differences in glycemia, however, all behavioral and electrophysiological parameters defining neuropathy remained identical in both models at 24 and 36 weeks. These data further support the contention that glucotoxicity alone does not drive the development and progression of neuropathy, even when present early in life.

Recent data strongly suggest that dyslipidemia significantly contributes to neuropathy (Feldman et al., 2017). This idea is supported by our recent findings in the SEARCH cohort where neuropathy in youth correlates with dyslipidemia (Guilford et al., 2011). To better understand the role of lipids in neuropathy, we examined plasma cholesterol profiles, acknowledging the caveat that dyslipidemia manifests differently in rodents and humans (Yin et al., 2012). We observe increases in cholesterol, LDL and oxLDL in both DIO and DIO-STZ mice with neuropathy, with comparable LDL levels to those of the SEARCH participants with neuropathy (Jaiswal et al., 2017). Elevated oxLDL levels are usually observed in type 2 diabetes (Feldman et al., 2017), and we have previously shown a role for oxLDL in neuropathy (Vincent et al., 2009). Interestingly, oxLDL levels were even higher in DIO-STZ mice than in DIO mice, yet had no additive effect on neuropathy, suggesting that even mild increases in oxLDL levels may be sufficient to induce neuropathy. A recent study also shows that lipid-binding liver X receptors, which are expressed on sensory neurons in DIO mice and regulate cholesterol and fatty acid homeostasis, play a role in the development of tactile allodynia, an early event in neuropathy pathogenesis (Gavini et al., 2018). These findings highlight the importance of performing earlier assessments of dyslipidemia and neuropathy to identify what specific factors contribute to disease. Collectively, our observations show that DIO and DIO-STZ mice develop similar degrees of neuropathy, despite markedly different degrees of hyperglycemia, supporting a role for altered lipid metabolism in neuropathy.

There is no ideal murine model for most human diseases. In the current study, instead of recapitulating the hyperinsulinemia typically seen in type 2 diabetes, DIO-STZ mice instead develop hypoinsulinemia, which is attributed to STZ-induced loss of insulin-producing β-cells. However, the pathogenesis of type 2 diabetes is different in youth versus adult patients, with younger patients tending to be insulin deficient rather than insulin resistant (Skovsø, 2014; Gungor et al., 2005). This feature makes young DIO-STZ mice a more suitable model of type 2 diabetes than mice administered STZ later in life, and highlights the importance of choosing the correct age of rodents when modeling disease.

We also acknowledge that we did not include a SD-STZ control group to determine whether STZ directly damages tissues other than the pancreas, including peripheral nerve tissue. The large doses of STZ used to model type 1 diabetes are known to have a pronounced effect on mice, which exhibit significant weight loss due to muscle atrophy (Deeds et al., 2011). As no significant weight loss was observed at any of the time points measured in the current study, we do not anticipate that the low dose of STZ we use had a noticeable direct effect on the peripheral nerve. Interestingly, however, the same DIO-STZ protocol used in more mature animals results in a loss of ∼10 g body weight (Yorek et al., 2015). Finally, as our aim in this study was to investigate neuropathy development using juvenile models of prediabetes and type 2 diabetes, induction of prediabetes and type 2 diabetes occurred when the mice were at an age considered to be adolescent and continued until 36 weeks, which is akin to young adulthood. Neuropathy was assessed longitudinally using hindpaw latency and sensory and motor NCVs, while IENFD measures were performed at study end to assess nerve structure. In our paradigm, the earliest time point for phenotyping mice began at 16 weeks of age, 11 weeks after HF feeding began; future studies of DIO and DIO-STZ mice could determine when neuropathy first appears as defined by behavior, electrophysiology and anatomical structure. We did not evaluate pain behaviors, another parameter that could be measured in future work, especially in light of a recent report demonstrating that, after 10 weeks of HF feeding, mice develop pain hypersensitivity before degeneration of small nerve fibers (Gavini et al., 2018).

In conclusion, we show that DIO and DIO-STZ mice develop metabolic changes and neuropathy in a manner that closely reflects disease progression in adolescents to adults with prediabetes and diabetes, respectively. While DIO-STZ mice display a similar degree of neuropathy as DIO mice, they develop hyperglycemia, making them more representative of frank diabetes. Despite having elevated glucose levels, however, DIO and DIO-STZ mice had nearly identical disease development, further suggesting that factors other than hyperglycemia may play a role in neuropathy progression. Hence, it is possible that dyslipidemia and/or altered insulin signaling resulting from obesity may contribute to nerve pathology. Finally, our study provides new juvenile murine models to assess the neurological complications that commonly afflict children and adolescents with obesity, prediabetes and diabetes. These preclinical disease models are needed to understand disease pathogenesis and develop mechanism-based therapies for neuropathy to allow early intervention in humans from youth to adulthood.

## MATERIALS AND METHODS

### Animals

Thirty male C57BL/6J mice (#000664, The Jackson Laboratory, Bar Harbor, ME, USA) were purchased at 4 weeks of age and housed in a specific pathogen-free suite kept at a temperature of 20±2°C with a 12:12 h light:dark cycle. Mice were housed five per cage with fine corncob bedding, provided nestlet as enrichment, and kept with littermates to avoid fighting and aggressive behavior. The animals had *ad libitum* access to drinking water and food. After 1 week of acclimation, cages were randomly allocated and mice were provided with a SD or HF diet. The SD contains 10% energy from fat, 70% energy from carbohydrates and 20% energy from protein (D12450B, Research Diets, Inc., New Brunswick, USA), while the HF diet contains 60% energy from fat, 20% energy from carbohydrates and 20% energy from protein (D12492, Research Diets, Inc., New Brunswick, USA). For all metabolic and neuropathy assessments, mice were transported into designated procedure rooms within the animal facility. All assessments were performed during the day between the hours of 9:00 a.m.-5:00 p.m. All procedures complied with Diabetic Complications Consortium (www.diacomp.org) protocols and were approved by the University of Michigan Institutional Animal Care and Use Committee (IACUC). At study conclusion, mice were euthanized with 150 mg/kg body weight of pentobarbital (Fatal-plus, Vortech Pharmaceuticals, Dearborn, MI, USA) delivered by intraperitoneal (IP) injection.

### Induction of type 2 diabetes

STZ was administered following the protocol described by the Diabetic Complications Consortium using the dosages described by Yorek et al. (2015). At 12 weeks, DIO mice were fasted (4 h fast) and body weights were recorded to determine appropriate dosing. Ten mice (two cages) were returned to the housing room while the remainder (who had similar body weight and fasting glucose) were transferred into a designated biohazard facility for administration of STZ. STZ was freshly prepared by making a 7.5 mg/ml solution in sodium citrate buffer (1.47 g Na_3_C_6_H_5_O_7_ per 50 ml ddH_2_O, pH 4.5). Mice were administered with 75 mg/kg body weight STZ solution (IP injection) using 1 ml disposable insulin syringes (Cat# 329420, BD Biosciences) in a fume hood. Once administered, mice were transferred back to their cages containing feed and, 72 h later, mice were injected with a second dose of STZ (50 mg/kg body weight STZ). To aid recovery post-STZ injection, animals were provided with DietGel (Cat# 72-06-5022, ClearH_2_O, Westbrook, ME, USA), while drinking water was supplemented with sucrose (10%) for 2 days after STZ injections to prevent hypoglycemic shock. No adverse events were observed in STZ-injected mice during the course of the study.

### Metabolic phenotyping

Fasting blood glucose levels were measured every 4 weeks from 8 weeks of age to document the development and duration of hyperglycemia. Following a 4 h fasting period, one drop of tail blood was analyzed using a glucometer (AlphaTrak, Abbott Laboratories, Inc., Alameda, CA, USA) with compatible glucose test strips (Zoetis, Dublin, Ireland). For GTT and ITT, 1 g/kg body weight glucose (Cat# G-8270, Sigma) or 0.075 U/kg body weight insulin (Novolin 70/30, Novo Nordisk, Plainsboro, NJ), respectively, was injected IP and blood glucose levels were serially recorded at 15, 30, 60 and 120 s. At the end of the experimental period, mice were fasted, euthanized and plasma was isolated for further testing. Mice injected with insulin who showed signs of hypoglycemic shock were rescued with 20% glucose solution (IP injection). Plasma insulin levels were assessed at the Mouse Metabolic Phenotyping Center (MMPC) at Vanderbilt (Vanderbilt, University School of Medicine, TN, USA), while cholesterol and triglyceride lipoprotein profiles were assessed by FPLC analysis at the MMPC located in Cincinnati (University of Cincinnati Medical Center, OH, USA; www.mmpc.org). OxLDL was measured via ELISA (CSB-E07933 m, Houston, TX, USA). All procedures complied with the Diabetic Complications Consortium (www.diacomp.org) protocols and were approved by the University of Michigan IACUC.

### Neuropathy phenotyping

Neuropathy phenotyping was performed as previously described (Russell et al., 1999; Sullivan et al., 2007; Oh et al., 2010) in accordance with guidelines provided by the Diabetic Complications Consortium (www.diacomp.org). Hindpaw withdrawal latency and sural sensory and sciatic motor NCVs were measured at 16, 24 and 36 weeks, while IENFD was determined at 36 weeks.

#### Hindpaw withdrawal latency

Thermal hindpaw withdrawal latency was measured using an analgesia meter (Model 336TG Life Sciences, Woodland Hills, CA, USA). Mice were placed in acrylic compartments on a warm (32°C) glass plate and allowed to habituate for 45 min. The light source was maneuvered under the hindpaw and the time of activation of the beam to the time of paw withdrawal was recorded (Lee et al., 1990). The light source was set at 25°C and the temperature increased to 55°C over the course of 20 s. A maximum threshold time limit of 30 s was applied to prevent injury to the mice. Hindpaw stimuli alternated between each foot, with approximately 10 min between each individual stimulus. Six measurements were taken per mouse and an average response time was calculated.

#### Nerve conduction studies

Mice were anesthetized using isoflurane (Hospira, Inc., Lake Forest, IL, USA) with a dose of 4-5% for induction and 1-2% for maintenance (Oh et al., 2010). Onset of anesthesia was judged by diminished righting reflex and decreased pedal withdrawal. The core temperature was maintained at 34°C with a heating lamp. The stainless-steel needle electrodes (Natus Biomedical, Madison, WI, USA) were cleaned with 70% alcohol between animals. Sural sensory NCV was determined by recording at the dorsum of the foot and antidromically stimulating with supramaximal stimulation at the ankle. NCV was calculated by dividing the distance by the take-off latency of the sensory nerve action potential. Sciatic-tibial motor NCV was determined by recording at the dorsum of the foot and orthodromically stimulating with supramaximal stimulation first at the ankle, then at the sciatic notch. Latencies were measured in each case from the initial onset of the compound muscle action potential. The motor NCV was calculated by subtracting the measured ankle distance from the measured notch distance. The resultant distance was then divided by the difference in the ankle and notch latencies for a final NCV.

#### Intraepidermal nerve fiber counts

Prior to perfusion, foot pads were collected from the plantar surface of the hindpaw, immersed in Newcomer Zamboni's fixative (Middletown, WI, USA) overnight at 4°C, rinsed in 5, 10 and 20% sucrose in 0.1 M sodium phosphate buffer, cryoembedded, sectioned (30 μm), and processed for pan-axonal marker, PGP9.5, immunofluorescence (Cat# 14730-1-ap, Batch# 00043233, 1:2000 Proteintech, Rosemont, IL, USA) (Polydefkis et al., 2001). Three images per mouse (3 mm) were collected on an Olympus FluoView 500 confocal microscope using a 20×1.2 objective at a resolution of 1024×1024 pixels. The optical section thickness was 3.3 μm. Ten images per stack were flattened using max project arithmetic option in MetaMorph (version 7.7.0.00). Counts and distances were added together and the data are presented as the number of fibers per millimeter (Christianson et al., 2003).

### Statistical analysis

Based on previously published studies (Vincent et al., 2009; Hinder et al., 2017), our reported sample sizes of *n*=8/group is adequately powered to detect significant differences. Analyses were performed using GraphPad Prism 7 (GraphPad Software, La Jolla, CA, USA), according to Festing and Altman (2002). Normality of data was determined using Brown–Forsythe *F*-tests. For normally distributed data, statistically significant differences (*P*<0.05) were determined using one-way ANOVA with Tukey's post-test for multiple comparisons. For non-normally distributed data, datasets were log2 transformed and the Brown–Forsythe *F*-test re-run. When log2 transformation normalized distribution, a one-way ANOVA with Tukey's post-test for multiple comparisons was run. When log2 transformation did not normalize distribution, the non-parametric Kruskal–Wallis test with Dunn's post-test for multiple comparisons was run on the original, non-transformed dataset. Data are presented as mean±standard error of the mean (s.e.m.) unless otherwise stated. The statistical methods for individual analyses and *P*-values for all dataset comparisons are provided in Tables S1-S15.

## Supplementary Material

Supplementary information
