# Supplementary material for: Juvenile murine models of prediabetes and type 2 diabetes develop neuropathy
Source: Dis Model Mech. 2018 Dec 18;11(12):dmm037374. doi: 10.1242/dmm.037374 (PMC6307897; doi:10.1242/dmm.037374)
Supplement: Supplementary information [file dmm-11-037374-s1.pdf]

Table S1. *Body Weight*

Group	Cage	Mouse ID	Weight (g)								
			8wk	12wk		16wk	20wk	23wk	24wk	32wk	36wk
			11.19.14	12.15.14		1.13.15	02.11.2015	03.02.2015	3.11.15	5.5.2015	05.29.2015
SD	AA	A	19.16	23.88		26.0	28.05	28.0	30.03	x	37.8
SD	AA	B	27.27	32.04		35.0	26.57	38.0	36.72	x	x
SD	AA	C	22.97	26.72		29.8	35.39	32.1	31.32	x	38.3
SD	AA	D	22.48	26.83		27.3	29.42	29.3	28.58	x	x
SD	AA	F	22.09	24.82		x	29.67	31.0	29.08	x	34.1
SD	BB	A	21.75	25.42		27.8	29.68	31.5	28.82	x	30.6
SD	BB	B	24.11	28.32		30.8	32.1	33.3	31.62	x	39.6
SD	BB	C	24.30	27.47		30.7	33.78	34.6	33.47	x	40.3
SD	BB	D	21.29	24.15		26.000	27.91	29.2	28.0	x	33.8
SD	BB	F	21.42	24.28		x	27.43	29.2	28.67	x	35.1
DIO	CC	A	29.44	40.36		49.5	39.61	43.0	41.74	x	49.4
DIO	CC	C	23.11	28.09		32.7	50.51	50.9	49.61	x	57.8
DIO	CC	E	24.57	30.06		35.1	50.31	51.6	49.49	x	59.2
DIO	DD	A	23.42	27.65		31.2	55.41	56.3	54.63	x	64.3
DIO	DD	C	26.8	37.22		46.1	38.81	43.0	38.68	x	51.4
DIO	DD	E	30.28	35.61		41.2	42.2	46.2	44.13	x	51.0
DIO	EE	B	23.4	26.52		29.2	37.87	43.1	39.09	x	50.8
DIO	EE	E	23.65	27.07		30.6	34.89	38.5	36.61	x	46.9
DIO	FF	B	26.88	35.47		x	51.58	51.2	51.5	x	46.9
DIO	FF	E	24.82	30.65		x	44.23	49.3	48.95	x	55.5
DIO-STZ	GG	A	23.75	30.08	STZ injection	34.6	40.63	43.7	43.03	50.2	54.8
DIO-STZ	GG	C	27.56	34.81		38.3	42.87	45.3	43.32	54	57.5
DIO-STZ	GG	E	23.61	28.19		29.6	34.26	35.9	36.30	35.1	43.2
DIO-STZ	HH	A	28.5	39.98		42.6	47.64	50.0	43.03	x	x
DIO-STZ	HH	C	26.08	36.85		39.5	44.43	45.7	43.65	52.3	56.0
DIO-STZ	HH	D	26.19	36.38		39.8	45.27	45.7	45.59	50	53.0
DIO-STZ	II	B	27.55	36.2		36.2	40.81	43.4	42.14	53	53.8
DIO-STZ	II	E	24.57	32.14		33.7	38.35	41.2	40.41	50.8	53.3
DIO-STZ	JJ	B	23.85	29.9		x	41.27	42.8	41.65	53.3	57.0
DIO-STZ	JJ	E	24.09	29.58		x	37.27	38.5	38.3	44.5	47.4

	Age (wk)	8	12	16	20	23	24	32	36
Ctrl	Average (g)	22.7	26.4	29.2	30.0	31.6	30.6	x	36.2
Ctrl	StDev	2.2	2.5	3.0	2.9	3.0	2.7	x	3.3
Ctrl	n	**10**	**10**	**8**	**10**	**10**	**10**	**x**	**8**
Ctrl	StError	0.691	0.791	1.076	0.912	0.962	0.867	x	1.181
Ctrl	Max threshold	27.05	31.39	35.26	35.77	37.70	36.11	x	42.90
Ctrl	Min threshold	18.31	21.39	23.09	24.23	25.54	25.15	x	29.54
DIO	Average (g)	25.6	31.9	37.0	44.5	47.3	45.4	x	53.3
DIO	StDev	2.6	4.9	7.7	7.0	5.4	6.2	x	5.7
DIO	n	**10**	**10**	**8**	**10**	**10**	**10**	**x**	**10**
DIO	StError	0.582	1.550	2.720	2.203	1.716	1.963	x	1.801
DIO	Max threshold	30.84	41.67	52.33	58.48	58.16	57.86	x	64.71
DIO	Min threshold	20.43	22.07	21.57	30.61	36.46	33.03	x	41.93
DIO-STZ	Average (g)	25.6	33.4	36.8	41.3	43.2	41.7	49.2	52.9
DIO-STZ	StDev	1.8	4.0	4.1	4.0	4.0	2.7	6.0	4.7
DIO-STZ	n	**10**	**10**	**8**	**10**	**10**	**10**	**9**	**9**
DIO-STZ	StError	0.581	1.249	1.457	1.262	1.260	0.867	2.003	1.569
DIO-STZ	Max threshold	29.25	41.31	45.03	49.26	51.19	47.23	61.26	62.28
DIO-STZ	Min threshold	21.90	25.51	28.55	33.30	35.25	36.26	37.22	43.46

Tukey's multiple comparisons test	Mean Diff.	95.00% CI of diff.	Significant?	Summary
8wk				
SD vs. HF	-2.953	-7.513 to 1.607	No	ns
SD vs. HF-STZ	-2.891	-7.451 to 1.669	No	ns
HF vs. HF-STZ	0.062	-4.498 to 4.622	No	ns
12wk				
SD vs. HF	-5.477	-10.04 to -0.9173	Yes	*
SD vs. HF-STZ	-7.018	-11.58 to -2.458	Yes	**
HF vs. HF-STZ	-1.541	-6.101 to 3.019	No	ns
16wk				
SD vs. HF	-7.775	-12.87 to -2.677	Yes	**
SD vs. HF-STZ	-7.613	-12.71 to -2.515	Yes	**
HF vs. HF-STZ	0.1625	-4.935 to 5.26	No	ns
20wk				
SD vs. HF	-14.54	-19.1 to -9.982	Yes	****
SD vs. HF-STZ	-11.28	-15.84 to -6.72	Yes	****
HF vs. HF-STZ	3.262	-1.298 to 7.822	No	ns
24wk				
SD vs. HF	-15.69	-20.25 to -11.13	Yes	****
SD vs. HF-STZ	-11.6	-16.16 to -7.04	Yes	****
HF vs. HF-STZ	4.09	-0.4697 to 8.65	No	ns
36wk				
SD vs. HF	-17.12	-21.96 to -12.28	Yes	****
SD vs. HF-STZ	-16.69	-21.64 to -11.73	Yes	****
HF vs. HF-STZ	0.4311	-4.253 to 5.116	No	ns

Table S2. ***Fasting Glucose***

Group	Cage	Mouse ID	Fasting glucose (mg/dL)					
			12wk	16wk	20wk	24wk	32wk	36wk
				01.14.2015	02.11.2015	03.02.2015	05.05.2015	05.29.2015
SD	AA	A		169	136	131	x	225
	AA	B		278	202	172	x	x
	AA	C		248	213	165	x	223
	AA	D		252	183	160	x	x
	AA	F		x	192	86	x	210
	BB	A		259	211	197	x	155
	BB	B		231	225	249	x	218
	BB	C		229	183	218	x	201
	BB	D		244	149	228	x	171
	BB	F		x	218	234	x	219
DIO	CC	A		402	251	309	x	212
	CC	C		270	260	242	x	244
	CC	E		259	258	283	x	214
	DD	A		309	248	177	x	221
	DD	C		405	299	241	x	287
	DD	E		345	278	281	x	273
	EE	B		193	218	276	x	238
	EE	E		254	229	258	x	270
	FF	B		x	272	302	x	154
	FF	E		x	224	324	x	222
DIO-STZ	GG	A	STZ injection	477	458	715	487	426
	GG	C		605	468	637	528	287
	GG	E		369	252	318	425	334
	HH	A		483	514	585	x	x
	HH	C		597	522	567	505	449
	HH	D		574	509	622	522	565
	II	B		440	438	545	367	371
	II	E		620	433	532	359	324
	JJ	B		x	627	574	613	475
	JJ	E		x	592	636	439	608

Tukey's multiple comparisons test	Mean Diff.	95.00% CI of diff.	Significant?	Summary
16.				
SD vs. HF	-65.88	-147.5 to 15.75	No	ns
SD vs. HF-STZ	-281.9	-363.5 to -200.2	Yes	****
HF vs. HF-STZ	-216	-297.6 to -134.4	Yes	****
20.				
SD vs. HF	-62.5	-135.5 to 10.51	No	ns
SD vs. HF-STZ	-290.1	-363.1 to -217.1	Yes	****
HF vs. HF-STZ	-227.6	-300.6 to -154.6	Yes	****
24.				
SD vs. HF	-85.3	-158.3 to -12.29	Yes	*
SD vs. HF-STZ	-389.1	-462.1 to -316.1	Yes	****
HF vs. HF-STZ	-303.8	-376.8 to -230.8	Yes	****
36.				
SD vs. HF	-30.75	-108.2 to 46.69	No	ns
SD vs. HF-STZ	-223.8	-303.1 to -144.5	Yes	****
HF vs. HF-STZ	-193.1	-268.1 to -118	Yes	****

	Age (wk)	16	20	24	32	36
Ctrl	Average (g)	238.8	191.2	184.0	x	202.8
	StDev	32.2	29.5	51.1	x	26.0
	n	8	10	10	x	8
	StError	11.4	9.3	16.2	x	9.2
	Max threshold	303.2	250.1	286.2	x	254.8
	Min threshold	174.3	132.3	81.8	x	150.7
DIO	Average (g)	304.6	253.7	269.3	x	233.5
	StDev	75.1	25.5	42.4	x	38.5
	n	8	10	10	x	10
	StError	26.562	8.057	13.416	x	15.715
	Max threshold	454.9	304.7	354.2	x	310.5
	Min threshold	154.4	202.7	184.4	x	156.5
DIO-STZ	Average (g)	520.6	481.3	573.1	471.7	426.6
	StDev	91.4	102.5	104.5	82.1	109.9
	n	8	10	10	9	7
	StError	32.3	32.4	33.0	27.4	41.5
	Max threshold	703.4	686.2	782.1	635.9	646.3
	Min threshold	337.8	276.4	364.1	307.5	206.8

Table S3. *Plasma Insulin*

Group	Mouse ID#	Insulin (ng/ml)
SD	3268	2.469
	3270	2.320
	3272	2.267
	3273	0.590
	3274	1.350
	3275	2.569
	3276	0.621
	3277	2.071
DIO	3280	10.440
	3281	11.594
	3282	8.211
	3283	7.498
	3284	9.827
	3285	11.610
	3286	0.729
	3287	4.133
DIO-STZ	3288	1.765
	3289	1.099
	3292	1.356
	3293	1.257
	3294	1.226
	3295	1.934
	3296	1.478
	3297	1.318
	3290	1.127

Group	SD	DIO	DIO-STZ
1	2.469	10.440	1.765
2	2.320	11.594	1.099
3	2.267	8.211	1.356
4	0.590	7.498	1.257
5	1.350	9.827	1.226
6	2.569	11.610	1.934
7	0.621	0.729	1.478
8	2.071	4.133	1.318
9	x	x	1.127
Average (g)	1.782	8.005	1.429
StDev	0.816	3.843	0.285
n	8	8	9
StError	0.288	1.359	0.095
Max threshold	3.41	15.69	2.00
Min threshold	0.15	0.32	0.86

Brown-Forsythe test	
F (DFn, DFd)	7.554 (2, 22)
P value	0.0032
P value summary	**
Are SDs significantly different (P < 0.05)?	Yes
⟶ log2 transform data and retest	
Brown-Forsythe test	
F (DFn, DFd)	1.157 (2, 22)
P value	0.3328
P value summary	ns
Are SDs significantly different (P < 0.05)?	No
⟶ Proceed with ordinary 1-way ANOVA	

Tukey's multiple comparisons test	Mean Diff.	95.00% CI of diff.	Significant?	Summary	Adjusted P Value
SD vs. DIO	-2.025	-3.189 to -0.8605	Yes	***	0.0007
SD vs. DIO-STZ	0.1804	-0.9508 to 1.312	No	ns	0.9157
DIO vs. DIO-STZ	2.205	1.074 to 3.336	Yes	***	0.0002

Disease Models & Mechanisms • Supplementary information

Table S4.

24wk Glucose Tolerance Testing									
	Animal ID	Tail Mark	BW (g)	Time (min)					
				0	15	30	60	120	180*
SD	AA	A	28	190	451	483	522	246	188
	AA	B	38	177	471	390	441	292	199
	AA	C	32.1	194	540	529	361	269	247
	AA	D	29.3	193	477	661	466	250	172
	AA	F	31	185	546	711	470	236	174
	BB	A	31.5	222	507	281	241	162	152
	BB	B	33.3	197	446	376	323	238	210
	BB	C	34.6	205	610	401	294	216	200
	BB	D	29.2	218	679	123	296	214	195
	BB	F	29.2	245	678	526	360	236	205
DIO	CC	A	43	316	710	750	750	565	257
	CC	C	50.9	327	591	715	590	365	214
	CC	E	51.6	357	706	750	750	619	320
	DD	A	56.3	309	349	319	367	269	249
	DD	C	43	296	750	750	750	750	750
	DD	E	46.2	428	632	750	739	373	229
	EE	B	43.1	341	643	727	740	493	267
	EE	E	38.5	298	379	622	710	572	265
	FF	B	51.2	296	661	728	651	539	244
	FF	E	49.3	378	611	750	750	580	267
DIO-STZ	GG	A	43.7	515	750	750	750	653	538
	GG	C	45.3	488	663	750	750	581	650
	GG	E	35.9	356	750	750	719	369	261
	HH	A	50	503	750	750	750	750	710
	HH	C	45.7	484	728	750	750	701	652
	HH	D	45.7	647	750	750	750	643	517
	II	B	43.4	423	750	750	750	689	611
	II	E	41.2	514	750	750	750	472	419
	JJ	B	42.8	719	750	750	750	726	670
	JJ	E	38.5	723	750	750	750	750	661

* An additional recording was taken at 180 min to ensure that glucose levels were returning to normal in mice with FG >750mg/dL

* Comparative statistics not performed as several recorded measures were above the level of detection (any readings >750 gave a reading of 'HI')

n	SD					DIO					DIO-STZ				
1	190	451	483	522	246	316	710	750	750	565	515	750	750	750	653
2	177	471	390	441	292	327	591	715	590	365	488	663	750	750	581
3	194	540	529	361	269	357	706	750	750	619	356	750	750	719	369
4	193	477	661	466	250	309	x	x	x	x	503	750	750	750	750
5	185	546	711	470	236	296	x	x	x	x	484	728	750	750	701
6	222	507	281	241	162	428	632	750	739	373	647	750	750	750	643
7	197	446	376	323	238	341	643	727	740	493	423	750	750	750	689
8	205	610	401	294	216	298	379	622	710	572	514	750	750	750	472
9	218	679	123	296	214	296	661	728	651	539	719	750	750	750	726
10	245	678	526	360	236	378	611	750	750	580	723	750	750	750	750
Mean	202.6	540.5	448.1	377.4	235.9	334.6	616.625	724	710	513.25	537.2	739.1	750	746.9	633.4
St.Dev	20.4	88.1	174.1	92.6	34.8	42.9	104.7	43.4	59.1	96.0	121.5	27.6	0.0	9.8	125.9
St.Error	6.4	27.9	58.0	30.9	12.3	13.6	33.1	13.7	18.7	30.4	38.4	8.7	0.0	3.1	39.8
Max threshold	243.4	716.7	796.2	562.5	305.5	420.3	826.1	810.9	828.1	705.2	780.3	794.3	750.0	766.5	885.2
Min threshold	161.8	364.3	100.0	192.3	166.3	248.9	407.2	637.1	591.9	321.3	294.1	683.9	750.0	727.3	381.6

0 min	Tukey's multiple comparisons test	Mean Diff.	95.00% CI of diff.	Significant?	Summary	Adjusted P Value			
	SD vs. DIO	-132	-215.5 to -48.47	Yes	**	0.0015	A-B		
	SD vs. DIO -STZ	-334.6	-418.1 to -251.1	Yes	****	<0.0001	A-C		
	DIO vs. DIO -STZ	-202.6	-286.1 to -119.1	Yes	****	<0.0001	B-C		
	Test details	Mean 1	Mean 2	Mean Diff.	SE of diff.	n1	n2	q	DF
	SD vs. DIO	202.6	334.6	-132	33.69	10	10	5.541	27
	SD vs. DIO -STZ	202.6	537.2	-334.6	33.69	10	10	14.05	27
	DIO vs. DIO -STZ	334.6	537.2	-202.6	33.69	10	10	8.505	27

15 min	Tukey's multiple comparisons test	Mean Diff.	95.00% CI of diff.	Significant?	Summary	Adjusted P Value			
	SD vs. DIO	-76.13	-168.7 to 16.46	No	ns	0.1216	A-B		
	SD vs. DIO -STZ	-198.6	-285.9 to -111.3	Yes	****	<0.0001	A-C		
	DIO vs. DIO -STZ	-122.5	-215.1 to -29.89	Yes	**	0.008	B-C		
	Test details	Mean 1	Mean 2	Mean Diff.	SE of diff.	n1	n2	q	DF
	SD vs. DIO	540.5	616.6	-76.13	37.17	10	8	2.896	25
	SD vs. DIO -STZ	540.5	739.1	-198.6	35.04	10	10	8.015	25
	DIO vs. DIO -STZ	616.6	739.1	-122.5	37.17	8	10	4.66	25

30 min	Tukey's multiple comparisons test	Mean Diff.	95.00% CI of diff.	Significant?	Summary	Adjusted P Value			
	SD vs. DIO	-275.9	-402.2 to -149.6	Yes	****	<0.0001	A-B		
	SD vs. DIO -STZ	-301.9	-421 to -182.8	Yes	****	<0.0001	A-C		
	DIO vs. DIO -STZ	-26	-152.3 to 100.3	No	ns	0.8659	B-C		
	Test details	Mean 1	Mean 2	Mean Diff.	SE of diff.	n1	n2	q	DF
	SD vs. DIO	448.1	724	-275.9	50.73	10	8	7.692	25
	SD vs. DIO -STZ	448.1	750	-301.9	47.82	10	10	8.928	25
	DIO vs. DIO -STZ	724	750	-26	50.73	8	10	0.7249	25

60 min	Tukey's multiple comparisons test	Mean Diff.	95.00% CI of diff.	Significant?	Summary	Adjusted P Value			
	SD vs. DIO	-332.6	-408.2 to -257	Yes	****	<0.0001	A-B		
	SD vs. DIO -STZ	-369.5	-440.8 to -298.2	Yes	****	<0.0001	A-C		
	DIO vs. DIO -STZ	-36.9	-112.5 to 38.71	No	ns	0.4553	B-C		
	Test details	Mean 1	Mean 2	Mean Diff.	SE of diff.	n1	n2	q	DF
	SD vs. DIO	377.4	710	-332.6	30.36	10	8	15.5	25
	SD vs. DIO -STZ	377.4	746.9	-369.5	28.62	10	10	18.26	25
	DIO vs. DIO -STZ	710	746.9	-36.9	30.36	8	10	1.719	25

120 min	Tukey's multiple comparisons test	Mean Diff.	95.00% CI of diff.	Significant?	Summary	Adjusted P Value			
	SD vs. DIO	-277.4	-387.7 to -167	Yes	****	<0.0001	A-B		
	SD vs. DIO -STZ	-397.5	-501.5 to -293.5	Yes	****	<0.0001	A-C		
	DIO vs. DIO -STZ	-120.2	-230.5 to -9.825	Yes	*	0.0309	B-C		
	Test details	Mean 1	Mean 2	Mean Diff.	SE of diff.	n1	n2	q	DF
	SD vs. DIO	235.9	513.3	-277.4	44.29	10	8	8.855	25
	SD vs. DIO -STZ	235.9	633.4	-397.5	41.76	10	10	13.46	25
	DIO vs. DIO -STZ	513.3	633.4	-120.2	44.29	8	10	3.836	25

Table S5.

24wk Insulin Tolerance Testing								
	Cage	Mouse	BW (g)	Time (min)				
				0	15	30	60	120
SD	AA	A	28	131	*42*	*125*	*66*	*113*
	AA	B	38	172	129	122	125	148
	AA	C	32.1	165	132	125	114	143
	AA	D	29.3	160	108	98	84	145
	AA	F	31	86	106	135	121	135
	BB	A	31.5	197	213	224	202	234
	BB	B	33.3	249	133	142	147	167
	BB	C	34.6	218	127	142	171	193
	BB	D	29.2	228	135	95	*24*	*136*
	BB	F	29.2	234	108	125	93	96
DIO	CC	A	43	309	265	279	274	233
	CC	C	50.9	242	258	230	240	172
	CC	E	51.6	283	383	376	299	262
	DD	A	56.3	177	212	192	191	185
	DD	C	43	241	263	248	228	251
	DD	E	46.2	281	243	274	208	232
	EE	B	43.1	276	312	264	253	228
	EE	E	38.5	258	216	205	212	209
	FF	B	51.2	302	295	260	234	239
	FF	E	49.3	324	309	246	215	256
DIO-STZ	GG	A	43.7	715	624	572	380	459
	GG	C	45.3	637	633	455	303	307
	GG	E	35.9	318	304	177	162	136
	HH	A	50	585	750	564	474	301
	HH	C	45.7	567	588	449	262	346
	HH	D	45.7	622	602	549	444	429
	II	B	43.4	545	653	605	666	567
	II	E	41.2	532	520	292	170	214
	JJ	B	42.8	574	585	429	284	502
	JJ	E	38.5	636	602	397	370	430

* Mice that developed hypoglycemia (<50mg/dL) were rescued with 20% glucose solution

n	SD					DIO					DIO-STZ				
1	131	42	x	x	x	309	265	279	274	233	715	624	572	380	459
2	172	129	122	125	148	242	258	230	240	172	637	633	455	303	307
3	165	132	125	114	143	283	383	376	299	262	318	304	177	162	136
4	160	108	98	84	145	177	212	192	191	185	585	750	564	474	301
5	86	106	135	121	135	241	263	248	228	251	567	588	449	262	346
6	197	213	224	202	234	281	243	274	208	232	622	602	549	444	429
7	249	133	142	147	167	276	312	264	253	228	545	653	605	666	567
8	218	127	142	171	193	258	216	205	212	209	532	520	292	170	214
9	228	135	95	24	x	302	295	260	234	239	574	585	429	284	502
10	234	108	125	93	96	324	309	246	215	256	636	602	397	370	430
Mean	184	123.3	134	120	158	269	276	257	235	227	573	586	449	352	369
St.Dev	51.1	41.9	37.7	51.7	41.3	42.4	51.2	50.4	32.7	29.7	104.5	115.2	135.2	151.8	133.2
St.Error	16.2	13.2	11.9	16.3	13.1	13.4	16.2	15.9	10.3	9.4	33.0	36.4	42.7	48.0	42.1
Max threshold	286.2	207.0	209.6	223.4	240.3	354.2	378.0	358.2	300.7	286.2	782.1	816.5	719.3	655.1	635.5
Min threshold	81.8	39.6	58.9	16.8	75.0	184.4	173.2	156.6	170.1	167.2	364.1	355.7	178.5	47.9	102.7

	Tukey's multiple comparisons test	Mean Diff.	95.00% CI of diff.	Significant?	Summary	Adjusted P Value			
0 min	SD vs. DIO	-85.3	-164.6 to -6.033	Yes	*	0.033	A-B		
	SD vs. DIO -STZ	-389.1	-468.4 to -309.8	Yes	****	<0.0001	A-C		
	DIO vs. DIO -STZ	-303.8	-383.1 to -224.5	Yes	****	<0.0001	B-C		
	Test details	Mean 1	Mean 2	Mean Diff.	SE of diff.	n1	n2	q	DF
	SD vs. DIO	184	269.3	-85.3	31.97	10	10	3.773	27
	SD vs. DIO -STZ	184	573.1	-389.1	31.97	10	10	17.21	27
	DIO vs. DIO -STZ	269.3	573.1	-303.8	31.97	10	10	13.44	27

	Tukey's multiple comparisons test	Mean Diff.	95.00% CI of diff.	Significant?	Summary	Adjusted P Value			
15 min	SD vs. DIO	-152.3	-237.3 to -67.26	Yes	***	0.0004	A-B		
	SD vs. DIO -STZ	-462.8	-547.8 to -377.8	Yes	****	<0.0001	A-C		
	DIO vs. DIO -STZ	-310.5	-395.5 to -225.5	Yes	****	<0.0001	B-C		
	Test details	Mean 1	Mean 2	Mean Diff.	SE of diff.	n1	n2	q	DF
	SD vs. DIO	123.3	275.6	-152.3	34.3	10	10	6.28	27
	SD vs. DIO -STZ	123.3	586.1	-462.8	34.3	10	10	19.08	27
	DIO vs. DIO -STZ	275.6	586.1	-310.5	34.3	10	10	12.8	27

	Tukey's multiple comparisons test	Mean Diff.	95.00% CI of diff.	Significant?	Summary	Adjusted P Value			
30 min	SD vs. DIO	-123.2	-223 to -23.38	Yes	*	0.0134	A-B		
	SD vs. DIO -STZ	-314.7	-414.5 to -214.9	Yes	****	<0.0001	A-C		
	DIO vs. DIO -STZ	-191.5	-288.6 to -94.36	Yes	***	0.0001	B-C		
	Test details	Mean 1	Mean 2	Mean Diff.	SE of diff.	n1	n2	q	DF
	SD vs. DIO	134.2	257.4	-123.2	40.16	9	10	4.337	26
	SD vs. DIO -STZ	134.2	448.9	-314.7	40.16	9	10	11.08	26
	DIO vs. DIO -STZ	257.4	448.9	-191.5	39.09	10	10	6.928	26

	Tukey's multiple comparisons test	Mean Diff.	95.00% CI of diff.	Significant?	Summary	Adjusted P Value			
60 min	SD vs. DIO	-115.3	-224.6 to -5.97	Yes	*	0.0373	A-B		
	SD vs. DIO -STZ	-231.4	-340.7 to -122.1	Yes	****	<0.0001	A-C		
	DIO vs. DIO -STZ	-116.1	-222.5 to -9.696	Yes	*	0.0305	B-C		
	Test details	Mean 1	Mean 2	Mean Diff.	SE of diff.	n1	n2	q	DF
	SD vs. DIO	120.1	235.4	-115.3	43.99	9	10	3.706	26
	SD vs. DIO -STZ	120.1	351.5	-231.4	43.99	9	10	7.438	26
	DIO vs. DIO -STZ	235.4	351.5	-116.1	42.82	10	10	3.834	26

	Tukey's multiple comparisons test	Mean Diff.	95.00% CI of diff.	Significant?	Summary	Adjusted P Value			
120 min	SD vs. DIO	-69.08	-169.2 to 31.05	No	ns	0.2182	A-B		
	SD vs. DIO -STZ	-211.5	-311.6 to -111.4	Yes	****	<0.0001	A-C		
	DIO vs. DIO -STZ	-142.4	-236.8 to -48	Yes	**	0.0026	B-C		
	Test details	Mean 1	Mean 2	Mean Diff.	SE of diff.	n1	n2	q	DF
	SD vs. DIO	157.6	226.7	-69.08	40.2	8	10	2.43	25
	SD vs. DIO -STZ	157.6	369.1	-211.5	40.2	8	10	7.44	25
	DIO vs. DIO -STZ	226.7	369.1	-142.4	37.9	10	10	5.314	25

Table S6. *Plasma Cholesterol*

Group	Mouse ID#	Cholesterol (mg/dL)
SD	3268	125.356
	3270	169.516
	3272	103.989
	3274	135.328
	3275	169.516
	3276	133.903
DIO	3280	226.496
	3281	327.635
	3282	242.165
	3283	213.675
	3284	195.157
	3285	192.308
DIO-STZ	3288	233.618
	3290	139.601
	3292	202.279
	3294	198.006
	3295	207.977
	3296	215.100

Group	SD	DIO	DIO-STZ
1	125.356	226.496	233.618
2	169.516	327.635	139.601
3	103.989	242.165	202.279
4	135.328	213.675	198.006
5	169.516	195.157	207.977
6	133.903	192.308	215.100
Average (g)	139.601	232.906	199.430
StDev	25.736	50.087	31.866
n	6	6	6
StError	10.507	20.448	13.009
Max threshold	191.07	333.08	263.16
Min threshold	88.13	132.73	135.70

Brown-Forsythe test	
F (DFn, DFd)	0.4761 (2, 15)
P value	0.6303
P value summary	ns
Are SDs significantly different (P < 0.05)?	No
→ Proceed with ordinary 1-way ANOVA	

Tukey's multiple comparisons test	Mean Diff.	95.00% CI of diff.	Significant?	Summary	Adjusted P Value
SD vs. DIO	-93.3	-149.3 to -37.28	Yes	**	0.0016
SD vs. DIO-STZ	-59.83	-115.9 to -3.808	Yes	*	0.0357
DIO vs. DIO-STZ	33.48	-22.55 to 89.5	No	ns	0.296

Disease Models & Mechanisms • Supplementary information

Table S7. *FPLC Cholesterol*

Fraction	SD						DIO						DIO-STZ					
1	0.22	0.216	0.073	0	0	0	0.224	0.076	0.149	0	0	0.073	0	0	0	0	0.08	0
2	0	0.144	0	0	0	0	0.075	0.076	0.074	0	0	0.073	0.075	0	0	0	0.08	0
3	0	0	0.146	0	0.073	0.075	0	0	0	0.076	0.15	0	0.075	0	0.075	0	0	0.077
4	0	0.144	0.146	0	0.147	0.15	0	0.076	0.074	0.076	0.075	0	0.451	0	0.075	0	0	0.077
5	0.22	0.144	0.51	0.292	0.147	0.224	0	0.076	0	0.076	0.075	0.145	0.15	0	0	0	0	0.077
6	0.073	0	0.219	0.073	0.147	0.15	0	0.076	0	0.076	0.075	0.508	0.075	0	0.075	0	0	0.154
7	0.147	0.072	0.073	0.219	0	0.075	0.075	0.076	0.074	0	0	0	0	0	0	0	0	0.462
8	0.073	0.072	0.073	0.146	0	0	0.075	0.076	0.074	0	0	0	0.075	0	0	0	0.08	0
9	0.073	0.216	0.073	0	0	0	0.224	0.152	0.074	0.076	0.075	0	0.075	0	0.075	0	0	0.308
10	0	0.072	0.146	0	0	0	0	0.076	0	0.076	0.075	0	0.075	0	0.075	0.078	0.08	0.154
11	0	0	0.146	0	0.22	0.15	0	0.227	0.074	0.076	0.075	0	0.075	0	0.075	0.157	0	0.231
12	0	0.144	0.292	0.146	0.147	0.15	0.075	0.682	0.074	0.227	0.15	0.073	0.226	0	0.301	0.313	0	0.693
13	0.147	0.144	0.219	0.219	0.22	0.15	0.149	0.985	0.074	0.302	0.15	0.073	0.376	0	0.301	0.391	0	0.616
14	0.294	0.289	0.292	0.292	0.293	0.374	0.671	1.667	0.223	0.529	0.301	0.145	0.677	0	0.602	0.705	0.161	0.924
15	0.881	0.722	0.437	0.585	0.513	0.524	0.97	2.728	0.744	0.907	0.451	0.29	1.053	0.227	0.752	1.018	0.562	1.386
16	1.396	1.01	0.729	1.242	0.879	0.748	1.641	4.168	0.967	1.361	0.677	0.508	1.579	0.531	1.353	1.644	1.445	2.002
17	1.763	1.443	1.02	1.315	1.245	1.047	2.163	5.002	1.414	2.192	0.977	0.653	2.18	0.606	1.805	2.192	1.445	2.618
18	1.91	1.732	1.312	1.535	1.612	1.496	2.686	5.911	1.86	3.175	1.429	1.015	3.308	0.909	2.481	2.896	1.926	3.851
19	1.983	2.02	1.458	1.973	2.198	1.87	3.655	7.351	3.125	4.233	2.105	2.03	4.361	1.516	3.308	3.757	2.408	5.391
20	2.057	2.597	1.458	2.338	2.93	2.244	5.073	9.473	4.762	5.896	3.308	2.538	5.865	2.425	5.038	4.149	3.531	7.162
21	1.983	3.03	1.312	2.631	3.297	2.393	6.789	11.898	6.473	8.163	4.887	3.771	7.669	3.41	6.391	5.245	5.056	8.086
22	2.35	3.896	1.312	2.996	4.249	2.767	9.026	14.778	8.929	10.431	6.541	5.076	9.248	4.396	8.045	6.106	6.742	9.472
23	2.718	4.545	1.166	2.996	4.762	3.067	10.593	16.521	10.417	12.018	8.12	6.019	10.15	4.926	8.947	6.967	7.945	10.089
24	3.158	5.195	1.166	3.581	4.982	3.141	11.936	18.037	11.979	11.943	9.474	6.526	10.602	5.532	10.15	7.436	9.47	10.32
25	3.085	5.483	1.239	2.923	4.982	3.141	11.712	17.507	11.756	11.338	9.474	7.107	10.451	4.85	9.925	7.906	8.909	9.395001
26	2.864	5.916	1.312	2.85	5.275	3.366	11.115	15.915	10.938	10.506	9.699	7.034	10.827	4.471	10.226	7.828	8.587	8.625
27	3.158	7.143	1.749	3.215	5.861	3.366	11.637	14.627	11.756	10.355	10.075	7.252	11.128	4.623	10.827	8.376	9.23	8.394
28	4.113	9.38	2.697	4.019	7.253	4.338	12.383	14.703	12.426	11.111	11.203	8.484	11.729	5.836	12.03	9.159	11.316	10.012
29	6.023	11.111	4.738	6.284	9.597	6.657	12.682	14.551	13.69	12.32	11.955	9.5	12.18	8.867	13.233	10.411	12.681	11.783
30	7.418	12.049	7.289	8.696	11.429	9.349	13.428	15.081	15.402	13.379	12.857	10.587	13.308	11.292	14.662	11.507	14.928	13.323
31	8.961	13.781	9.548	10.888	13.7	12.042	16.561	18.568	20.015	15.571	16.466	11.82	15.038	15.46	18.722	13.151	18.539	16.25
32	12.707	17.027	13.921	16.588	18.388	17.053	23.275	25.085	26.339	18.821	22.707	13.27	17.669	23.191	23.91	16.204	26.003	20.87
33	17.113	19.841	20.117	20.168	22.418	22.289	28.497	28.799	30.06	18.443	24.887	13.488	20	25.692	25.564	17.691	26.726	24.182
34	17.407	18.831	20.845	20.022	19.78	20.942	25.289	26.753	23.958	20.257	21.654	17.331	21.353	20.235	20.301	19.178	19.502	19.715
35	10.87	11.111	13.557	11.911	11.282	12.191	14.472	14.551	12.054	10.506	11.579	10.95	11.729	9.094	10.226	10.568	9.631	9.935
36	4.48	4.69	5.831	4.384	4.542	4.862	5.446	5.457	4.241	3.704	4.286	4.061	10.376	3.107	3.985	3.914	3.21	3.774
37	1.396	1.515	1.968	1.388	1.392	1.496	1.492	1.592	1.265	1.285	1.429	1.378	1.429	0.909	1.353	1.331	0.803	1.309
38	0.441	0.577	0.656	0.731	0.513	0.524	0.522	0.682	0.521	0.454	0.526	0.508	0.526	0.303	0.602	0.47	0.321	0.231
39	0.073	0.216	0.292	0.146	0.22	0.224	0.149	0.227	0.149	0.68	0.451	0.363	0.376	0.152	0.376	0.313	0	0.385
40	0.734	0.649	0.219	1.023	0.293	0.299	1.119	0.682	0.818	0.227	0.526	0.218	0.226	0.758	0.451	0.235	1.284	0.385
41	0.367	0.505	0.146	0.219	0.147	0.15	0.522	0.379	0.446	0.227	0.226	0.29	0.226	0.227	0.301	0.548	0.321	0.308
42	0	0.072	0.146	0	0.147	0.15	0	0.076	0.074	0.151	0.226	0.145	0.15	0	0.15	0.078	0	0.231
43	0	0.289	0.292	0.073	0.22	0.15	0	0.076	0.074	0.076	0.226	0.073	0.075	0	0.15	0.078	0	0.231
44	0	0.072	0.219	0	0.22	0.075	0	0	0	0.076	0.075	0.073	0.075	0	0.075	0.078	0	0.154
45	0	0.072	0.219	0	0.147	0.15	0	0.076	0	0.076	0.15	0.073	0.075	0	0.15	0.078	0	0.154
46	0	0.144	0.146	0.292	0.147	0.15	0	0.076	0.074	0.076	0.15	0.073	0.075	0	0.075	0.078	0	0.077
47	0	0	0.073	0	0	0	0	0	0	0.076	0.075	0	0.075	0	0.075	0	0	0.077
48	0.808	0.505	0.073	1.096	0.147	0.15	1.194	0.682	0.818	0	0.301	0	0	0.758	0.226	0	0	0.154
49	0.073	0.144	0	0.073	0	0	0.224	0.076	0.149	0	0.15	0	0	0	0	0	0	0
50	0	0	0.073	0	0	0	0	0	0	0.076	0.075	0	0	0	0.075	0	0	0.077

	SD				DIO				DIO-STZ			
Fraction	Mean	StDev	n	StdError	Mean	StDev	n	StdError	Mean	StDev	n	StdError
1	0.085	0.107	6	0.044	0.087	0.087	6	0.036	0.013	0.033	6	0.013
2	0.024	0.059	6	0.024	0.050	0.038	6	0.016	0.026	0.040	6	0.016
3	0.049	0.060	6	0.024	0.038	0.063	6	0.026	0.038	0.041	6	0.017
4	0.098	0.076	6	0.031	0.050	0.039	6	0.016	0.101	0.176	6	0.072
5	0.256	0.136	6	0.056	0.062	0.055	6	0.022	0.038	0.063	6	0.026
6	0.110	0.077	6	0.031	0.123	0.192	6	0.079	0.051	0.063	6	0.026
7	0.098	0.075	6	0.031	0.038	0.041	6	0.017	0.077	0.189	6	0.077
8	0.061	0.055	6	0.022	0.038	0.041	6	0.017	0.026	0.040	6	0.016
9	0.060	0.084	6	0.034	0.100	0.077	6	0.032	0.076	0.119	6	0.049
10	0.036	0.061	6	0.025	0.038	0.041	6	0.017	0.077	0.049	6	0.020
11	0.086	0.098	6	0.040	0.075	0.083	6	0.034	0.090	0.091	6	0.037
12	0.147	0.092	6	0.038	0.214	0.238	6	0.097	0.256	0.256	6	0.105
13	0.183	0.040	6	0.016	0.289	0.351	6	0.143	0.281	0.242	6	0.099
14	0.306	0.034	6	0.014	0.589	0.563	6	0.230	0.512	0.354	6	0.145
15	0.610	0.163	6	0.067	1.015	0.879	6	0.359	0.833	0.409	6	0.167
16	1.001	0.271	6	0.111	1.554	1.348	6	0.550	1.426	0.492	6	0.201
17	1.306	0.276	6	0.113	2.067	1.565	6	0.639	1.808	0.710	6	0.290
18	1.600	0.206	6	0.084	2.679	1.772	6	0.723	2.562	1.046	6	0.427
19	1.917	0.249	6	0.102	3.750	1.963	6	0.801	3.457	1.381	6	0.564
20	2.271	0.501	6	0.204	5.175	2.434	6	0.994	4.695	1.696	6	0.692
21	2.441	0.721	6	0.295	6.997	2.848	6	1.163	5.976	1.758	6	0.718
22	2.928	1.064	6	0.434	9.130	3.370	6	1.376	7.335	1.961	6	0.801
23	3.209	1.317	6	0.538	10.615	3.584	6	1.463	8.171	2.011	6	0.821
24	3.537	1.467	6	0.599	11.649	3.796	6	1.550	8.918	2.014	6	0.822
25	3.476	1.541	6	0.629	11.482	3.453	6	1.410	8.573	2.022	6	0.826
26	3.597	1.707	6	0.697	10.868	2.889	6	1.179	8.427	2.239	6	0.914
27	4.082	2.005	6	0.818	10.950	2.426	6	0.991	8.763	2.345	6	0.957
28	5.300	2.498	6	1.020	11.718	2.047	6	0.836	10.014	2.319	6	0.947
29	7.402	2.424	6	0.990	12.450	1.730	6	0.706	11.526	1.616	6	0.660
30	9.372	2.001	6	0.817	13.456	1.733	6	0.708	13.170	1.527	6	0.623
31	11.487	2.048	6	0.836	16.500	2.807	6	1.146	16.193	2.146	6	0.876
32	15.947	2.162	6	0.882	21.583	4.811	6	1.964	21.308	3.791	6	1.548
33	20.324	1.942	6	0.793	24.029	6.665	6	2.721	23.309	3.625	6	1.480
34	19.638	1.338	6	0.546	22.540	3.477	6	1.419	20.047	0.770	6	0.315
35	11.820	0.986	6	0.402	12.352	1.754	6	0.716	10.197	0.905	6	0.369
36	4.798	0.533	6	0.218	4.533	0.741	6	0.302	4.728	2.791	6	1.140
37	1.526	0.224	6	0.091	1.407	0.125	6	0.051	1.189	0.263	6	0.107
38	0.574	0.105	6	0.043	0.536	0.077	6	0.031	0.409	0.145	6	0.059
39	0.195	0.076	6	0.031	0.337	0.207	6	0.084	0.267	0.158	6	0.064
40	0.536	0.318	6	0.130	0.598	0.350	6	0.143	0.557	0.406	6	0.166
41	0.256	0.149	6	0.061	0.348	0.121	6	0.050	0.322	0.118	6	0.048
42	0.086	0.073	6	0.030	0.112	0.079	6	0.032	0.102	0.092	6	0.038
43	0.171	0.119	6	0.048	0.088	0.074	6	0.030	0.089	0.089	6	0.037
44	0.098	0.100	6	0.041	0.037	0.041	6	0.017	0.064	0.058	6	0.024
45	0.098	0.089	6	0.036	0.063	0.056	6	0.023	0.076	0.068	6	0.028
46	0.147	0.092	6	0.038	0.075	0.047	6	0.019	0.051	0.039	6	0.016
47	0.012	0.030	6	0.012	0.025	0.039	6	0.016	0.038	0.041	6	0.017
48	0.463	0.417	6	0.170	0.499	0.481	6	0.196	0.190	0.294	6	0.120
49	0.048	0.059	6	0.024	0.100	0.090	6	0.037	0.000	0.000	6	0.000
50	0.012	0.030	6	0.012	0.025	0.039	6	0.016	0.025	0.039	6	0.016

Disease Models & Mechanisms • Supplementary information

Table S8. *Cholesterol - vLDL*

Group	Mouse ID#	vLDL Cholesterol (mg/dL)
SD	3268	0.820
	3270	1.230
	3272	1.627
	3274	0.850
	3275	0.897
	3276	0.905
DIO	3280	0.682
	3281	1.726
	3282	0.652
	3283	0.729
	3284	0.697
	3285	1.090
DIO-STZ	3288	1.390
	3290	0.000
	3292	0.668
	3294	0.595
	3295	0.313
	3296	2.143

Group	SD	DIO	DIO-STZ
1	0.820	0.682	1.390
2	1.230	1.726	0.000
3	1.627	0.652	0.668
4	0.850	0.729	0.595
5	0.897	0.697	0.313
6	0.905	1.090	2.143
Average (g)	1.055	0.929	0.852
StDev	0.317	0.423	0.784
n	6	6	6
StError	0.129	0.173	0.320
Max threshold	1.69	1.77	2.42
Min threshold	0.42	0.08	-0.72

Brown-Forsythe test	
F (DFn, DFd)	1.159 (2, 15)
P value	0.3405
P value summary	ns
Are SDs significantly different (P < 0.05)?	No
→ Proceed with ordinary 1-way ANOVA	

Tukey's multiple comparisons test	Mean Diff.	95.00% CI of diff.	Significant?	Summary	Adjusted P Value
SD vs. DIO	0.1256	-0.6928 to 0.944	No	ns	0.9166
SD vs. DIO-STZ	0.2032	-0.6152 to 1.022	No	ns	0.7979
DIO vs. DIO-STZ	0.0776	-0.7408 to 0.896	No	ns	0.9672

Disease Models & Mechanisms • Supplementary information

Table S9. *Cholesterol - LDL*

Group	Mouse ID#	LDL Cholesterol (mg/dL)
SD	3268	26.979
	3270	38.145
	3272	12.389
	3274	26.640
	3275	38.193
	3276	24.498
DIO	3280	71.453
	3281	136.626
	3282	71.737
	3283	79.992
	3284	53.370
	3285	53.570
DIO-STZ	3288	80.446
	3290	28.719
	3292	61.613
	3294	63.280
	3295	56.814
	3296	76.700

Group	SD	DIO	DIO-STZ
1	26.979	71.453	80.446
2	38.145	136.626	28.719
3	12.389	71.737	61.613
4	26.640	79.992	63.280
5	38.193	53.370	56.814
6	24.498	53.570	76.700
Average (g)	27.807	77.791	61.262
StDev	9.649	30.745	18.382
n	6	6	6
StError	3.939	12.552	7.504
Max threshold	47.10	139.28	98.03
Min threshold	8.51	16.30	24.50

Brown-Forsythe test	
F (DFn, DFd)	0.7793 (2, 15)
P value	0.4764
P value summary	ns
Are SDs significantly different (P < 0.05)?	No
→ Proceed with ordinary 1-way ANOVA	

Tukey's multiple comparisons test	Mean Diff.	95.00% CI of diff.	Significant?	Summary	Adjusted P Value
SD vs. DIO	-49.98	-82.1 to -17.86	Yes	**	0.0029
SD vs. DIO-STZ	-33.45	-65.57 to -1.335	Yes	*	0.0407
DIO vs. DIO-STZ	16.53	-15.59 to 48.65	No	ns	0.3976

Table S10. *Cholesterol - HDL*

Group	Mouse ID#	HDL Cholesterol (mg/dL)
SD	3268	97.557
	3270	130.141
	3272	89.973
	3274	107.837
	3275	130.425
	3276	108.501
DIO	3280	154.361
	3281	189.283
	3282	169.777
	3283	132.955
	3284	141.090
	3285	137.648
DIO-STZ	3288	151.782
	3290	110.883
	3292	139.998
	3294	134.130
	3295	150.850
	3296	136.257

Group	SD	DIO	DIO-STZ
1	97.557	154.361	151.782
2	130.141	189.283	110.883
3	89.973	169.777	139.998
4	107.837	132.955	134.130
5	130.425	141.090	150.850
6	108.501	137.648	136.257
Average (g)	110.739	154.186	137.317
StDev	16.628	21.775	14.897
n	6	6	6
StError	6.788	8.889	6.082
Max threshold	143.99	197.73	167.11
Min threshold	77.48	110.64	107.52

Brown-Forsythe test	
F (DFn, DFd)	0.7793 (2, 15)
P value	0.4764
P value summary	ns
Are SDs significantly different (P < 0.05)?	No
→ Proceed with ordinary 1-way ANOVA	

Tukey's multiple comparisons test	Mean Diff.	95.00% CI of diff.	Significant?	Summary	Adjusted P Value
SD vs. DIO	-43.45	-70.45 to -16.45	Yes	**	0.0022
SD vs. DIO-STZ	-26.58	-53.58 to 0.4235	No	ns	0.054
DIO vs. DIO-STZ	16.87	-10.13 to 43.87	No	ns	0.2669

Disease Models & Mechanisms • Supplementary information

Table S11. *Plasma oxLDL*

Group	Mouse ID#	oxLDL (ng/dL)
SD	3268	15.64
	3270	15.34
	3272	16.24
	3273	7.94
	3274	28.64
	3275	25.44
	3276	18.04
DIO	3280	39.24
	3281	40.74
	3282	44.74
	3283	35.14
	3284	43.14
	3285	47.74
DIO-STZ	3288	55.64
	3290	67.94
	3292	62.54
	3294	56.04
	3295	55.74
	3296	64.84

Brown-Forsythe test	
F (DFn, DFd)	0.2649 (2, 16)
P value	0.7706
P value summary	ns
Are SDs significantly different (P < 0.05)?	No
→ Proceed with ordinary 1-way ANOVA	

Tukey's multiple comparisons test	Mean Diff.	95.00% CI of diff.	Significant?	Summary	Adjusted P Value
SD vs. DIO	-23.61	-31.85 to -15.36	Yes	****	<0.0001
SD vs. DIO-STZ	-42.27	-50.52 to -34.03	Yes	****	<0.0001
DIO vs. DIO-STZ	-18.67	-27.22 to -10.11	Yes	***	0.0001

Group	SD	DIO	DIO-STZ
1	15.64	39.24	55.640
2	15.34	40.74	67.940
3	16.24	44.74	62.540
4	7.94	35.14	56.040
5	28.64	43.14	55.740
6	25.44	47.74	64.840
7	18.04	x	x
Average (g)	18.183	41.790	60.457
StDev	6.895	4.422	5.376
n	7	6	6
StError	2.606	1.805	2.195
Max threshold	31.97	50.63	71.21
Min threshold	4.39	32.95	49.70

Table S12. *Motor NCV*

Group	Cage	Mouse ID	Motor NCV (m/s) 16wk	24wk	36wk
SD	AA	3268	62.0	66.0	62.5
	AA	3269	64.0	64.7	60.0
	AA	3270	58.0	60.7	60.0
	AA	3271	64.5	64.0	60.7
	AA	3272	59.6	62.0	59.6
	BB	3273	60.0	62.7	56.1
	BB	3274	62.5	64.0	61.5
	BB	3275	63.2	62.7	60.0
	BB	3276	60.0	65.1	58.8
	BB	3277	63.2	62.0	60.0
DIO	CC	3278	x	47.4	46.6
	CC	3279	51.7	49.1	47.5
	CC	3280	51.6	51.6	50.0
	DD	3281	59.6	46.9	48.3
	DD	3282	51.7	45.7	44.2
	DD	3283	48.3	50	49.1
	EE	3284	49.1	45.5	50.0
	EE	3285	53.3	44.2	49.1
	FF	3286	52.5	50.8	50.0
	FF	3287	51.6	46.6	43.4
DIO-STZ	GG	3288	48.3	53.3	51.9
	GG	3289	51.6	44.2	44.2
	GG	3290	50	46	48.3
	HH	3291	53.3	44.4	x
	HH	3292	54.2	48.3	46.6
	HH	3293	50	47.5	46.8
	II	3294	52	47.6	49.2
	II	3295	50	52.5	49.1
	JJ	3296	x	49.1	45.0
	JJ	3297	x	51.6	50.0

	Age (wk)	16wk	24wk	36wk
SD	Average (g)	61.7	63.4	59.9
	StDev	2.166	1.641	1.691
	n	10	10	10
	StError	0.685	0.519	0.535
	Max threshold	66.0	66.7	63.3
	Min threshold	57.4	60.1	56.5
DIO	Average (g)	52.2	47.8	47.8
	StDev	3.2	2.5	2.4
	n	9	10	10
	StError	1.068	0.782	0.760
	Max threshold	58.6	52.7	52.6
	Min threshold	45.7	42.8	43.0
DIO-STZ	Average (g)	51.2	48.5	47.9
	StDev	2.0	3.2	2.5
	n	8	10	9
	StError	0.7	1.0	0.8
	Max threshold	55.1	54.9	52.8
	Min threshold	47.3	42.0	43.0

16wk	Brown-Forsythe test					
	F (DFn, DFd)	0.8018 (2, 23)				
	P value	0.4607				
	P value summary	ns				
	Are SDs significantly different (P < 0.05)?	No				
	→ Proceed with ordinary 1-way ANOVA					
	Tukey's multiple comparisons test	Mean Diff.	95.00% CI of diff.	Significant?	Summary	Adjusted P Value
	SD vs. DIO	10.48	8.139 to 12.81	Yes	****	<0.0001
	SD vs. DIO-STZ	10.53	8.189 to 12.86	Yes	****	<0.0001
	DIO vs. DIO-STZ	0.05	-2.412 to 2.512	No	ns	0.9986
24wk	Brown-Forsythe test					
	F (DFn, DFd)	1.562 (2, 27)				
	P value	0.2282				
	P value summary	ns				
	Are SDs significantly different (P < 0.05)?	No				
	→ Proceed with ordinary 1-way ANOVA					
	Tukey's multiple comparisons test	Mean Diff.	95.00% CI of diff.	Significant?	Summary	Adjusted P Value
	SD vs. DIO	15.61	12.81 to 18.41	Yes	****	<0.0001
	SD vs. DIO-STZ	14.94	12.14 to 17.74	Yes	****	<0.0001
	DIO vs. DIO-STZ	-0.67	-3.466 to 2.126	No	ns	0.8244
36wk	Brown-Forsythe test					
	F (DFn, DFd)	2.229 (2, 25)				
	P value	0.1286				
	P value summary	ns				
	Are SDs significantly different (P < 0.05)?	No				
	→ Proceed with ordinary 1-way ANOVA					
	Tukey's multiple comparisons test	Mean Diff.	95.00% CI of diff.	Significant?	Summary	Adjusted P Value
	SD vs. DIO	12.52	10.12 to 14.93	Yes	****	<0.0001
	SD vs. DIO-STZ	12.44	9.979 to 14.91	Yes	****	<0.0001
	DIO vs. DIO-STZ	-0.08	-2.483 to 2.323	No	ns	0.9962

Disease Models & Mechanisms • Supplementary information

Table S13. *Sensory NCV*

Group	Cage	Mouse ID	Sensory NCV (m/s)		
			16wk	24wk	36wk
SD	AA	3268	23.3	23.3	22.5
	AA	3269	23.3	22.5	22.5
	AA	3270	24.1	25	25.8
	AA	3271	23.3	23.7	22.5
	AA	3272	23	23.3	23.5
	BB	3273	23.3	23.3	23.3
	BB	3274	23.3	22.5	23.3
	BB	3275	20	24.1	22.5
	BB	3276	22.5	23.3	23.3
	BB	3277	23.3	25.8	23.3
DIO	CC	3278	x	18.4	18.9
	CC	3279	20	15	17.5
	CC	3280	19.3	20.5	17.9
	DD	3281	19.3	15.3	18.4
	DD	3282	19.3	15	16.6
	DD	3283	18.9	16.6	17.0
	EE	3284	17.5	18.1	17.0
	EE	3285	17.5	17	17.5
	FF	3286	17.1	16.2	18.4
	FF	3287	17.5	16.2	17.1
DIO-STZ	GG	3288	17.5	17	17.5
	GG	3289	17.5	17.5	18.9
	GG	3290	17	17.5	17.0
	HH	3291	17.5	15.9	x
	HH	3292	17.5	14	21.8
	HH	3293	19.3	17.5	17.0
	II	3294	19	17	17.5
	II	3295	19	17.9	17.9
	JJ	3296	x	16.6	16.6
	JJ	3297	x	17.5	17.5

	Age (wk)	16wk	24wk	36wk
SD	Average (g)	22.9	23.7	23.3
	StDev	1.104	1.042	0.990
	n	10	10	10
	StError	0.349	0.330	0.313
	Max threshold	25.1	25.8	25.2
	Min threshold	20.7	21.6	21.3
DIO	Average (g)	18.5	16.8	17.6
	StDev	1.1	1.7	0.7
	n	9	10	10
	StError	0.359	0.552	0.237
	Max threshold	20.6	20.3	19.1
	Min threshold	16.3	13.3	16.1
DIO-STZ	Average (g)	18.0	16.8	18.0
	StDev	0.9	1.2	1.6
	n	8	10	9
	StError	0.3	0.4	0.5
	Max threshold	19.8	19.1	21.1
	Min threshold	16.2	14.5	14.8

16wk	Brown-Forsythe test					
	F (DFn, DFd)	0.544 (2, 24)				
	P value	0.5874				
	P value summary	ns				
	Are SDs significantly different (P < 0.05)?	No				
	→ Proceed with ordinary 1-way ANOVA					
	Tukey's multiple comparisons test	Mean Diff.	95.00% CI of diff.	Significant?	Summary	Adjusted P Value
	SD vs. DIO	4.451	3.258 to 5.644	Yes	****	<0.0001
	SD vs. DIO-STZ	4.903	3.671 to 6.134	Yes	****	<0.0001
	DIO vs. DIO-STZ	0.4514	-0.81 to 1.713	No	ns	0.6494
24wk	Brown-Forsythe test					
	F (DFn, DFd)	0.239 (2, 26)				
	P value	0.7892				
	P value summary	ns				
	Are SDs significantly different (P < 0.05)?	No				
	→ Proceed with ordinary 1-way ANOVA					
	Tukey's multiple comparisons test	Mean Diff.	95.00% CI of diff.	Significant?	Summary	Adjusted P Value
	SD vs. DIO	7.258	5.948 to 8.567	Yes	****	<0.0001
	SD vs. DIO-STZ	6.84	5.566 to 8.114	Yes	****	<0.0001
	DIO vs. DIO-STZ	-0.4178	-1.727 to 0.8916	No	ns	0.7107
36wk	Brown-Forsythe test					
	F (DFn, DFd)	0.5478 (2, 24)				
	P value	0.5853				
	P value summary	ns				
	Are SDs significantly different (P < 0.05)?	No				
	→ Proceed with ordinary 1-way ANOVA					
	Tukey's multiple comparisons test	Mean Diff.	95.00% CI of diff.	Significant?	Summary	Adjusted P Value
	SD vs. DIO	5.337	4.593 to 6.08	Yes	****	<0.0001
	SD vs. DIO-STZ	5.479	4.693 to 6.265	Yes	****	<0.0001
	DIO vs. DIO-STZ	0.1425	-0.6248 to 0.9098	No	ns	0.8887

Disease Models & Mechanisms • Supplementary information

Table S14. *Hindpaw Latency*

Group	Cage	Mouse ID	Hindpaw latency (s)		
			16wk	24wk	36wk
SD	AA	3268	3.64	2.87	3.86
	AA	3269	3.03	3.13	3.66
	AA	3270	3.18	2.94	4.23
	AA	3271	3.86	3.60	x
	AA	3272	3.74	2.93	x
	BB	3273	3.02	x	3.53
	BB	3274	3.32	3.14	3.70
	BB	3275	3.18	3.28	3.78
	BB	3276	3.63	2.62	3.71
	BB	3277	3.74	3.09	3.69
DIO	CC	3278	3.35	4.54	4.73
	CC	3279	3.48	4.44	4.92
	CC	3280	3.66	4.76	3.94
	DD	3281	3.60	4.89	4.47
	DD	3282	4.22	3.87	4.38
	DD	3283	4.12	4.91	5.28
	EE	3284	3.32	4.53	4.91
	EE	3285	2.86	3.60	3.89
	FF	3286	2.92	3.99	5.22
	FF	3287	3.15	5.00	4.55
DIO-STZ	GG	3288	3.52	4.38	4.98
	GG	3289	2.72	3.65	4.57
	GG	3290	3.52	4.58	4.53
	HH	3291	3.60	4.42	6.40
	HH	3292	3.47	4.26	4.26
	HH	3293	3.09	4.28	x
	II	3294	2.69	4.07	5.55
	II	3295	2.66	4.21	4.52
	JJ	3296	2.87	3.46	4.57
	JJ	3297	2.74	3.91	4.62

	Age (wk)	16wk	24wk	36wk
SD	Average (g)	3.4	3.1	3.8
	StDev	0.322	0.278	0.209
	n	10	9	8
	StError	0.102	0.093	0.074
	Max threshold	4.1	3.62	4.19
	Min threshold	2.8	2.5	3.4
DIO	Average (g)	3.5	4.5	4.6
	StDev	0.5	0.5	0.5
	n	10	10	10
	StError	0.151	0.153	0.151
	Max threshold	4.4	5.4	5.6
	Min threshold	2.6	3.5	3.7
DIO-STZ	Average (g)	3.1	4.1	4.9
	StDev	0.4	0.4	0.7
	n	10	10	9
	StError	0.1	0.1	0.2
	Max threshold	3.9	4.8	6.2
	Min threshold	2.3	3.41	3.5

16wk	Brown-Forsythe test					
	F (DFn, DFd)	0.3192 (2, 27)				
	P value	0.7294				
	P value summary	ns				
	Are SDs significantly different (P < 0.05)?	No				
	→ Proceed with ordinary 1-way ANOVA					
	Tukey's multiple comparisons test	Mean Diff.	95.00% CI of diff.	Significant?	Summary	Adjusted P Value
	SD vs. DIO	-0.034	-0.4716 to 0.4036	No	ns	0.9798
	SD vs. DIO-STZ	0.346	-0.0916 to 0.7836	No	ns	0.1416
	DIO vs. DIO-STZ	0.38	-0.0576 to 0.8176	No	ns	0.098

24wk	Brown-Forsythe test					
	F (DFn, DFd)	1.091 (2, 26)				
	P value	0.3508				
	P value summary	ns				
	Are SDs significantly different (P < 0.05)?	No				
	→ Proceed with ordinary 1-way ANOVA					
	Tukey's multiple comparisons test	Mean Diff.	95.00% CI of diff.	Significant?	Summary	Adjusted P Value
	SD vs. DIO	-1.386	-1.825 to -0.947	Yes	****	<0.0001
	SD vs. DIO-STZ	-1.055	-1.493 to -0.6158	Yes	****	<0.0001
	DIO vs. DIO-STZ	0.3312	-0.0959 to 0.7583	No	ns	0.1513

36wk	Brown-Forsythe test					
	F (DFn, DFd)	3.207 (2, 22)				
	P value	0.0599				
	P value summary	ns				
	Are SDs significantly different (P < 0.05)?	No				
	→ Proceed with ordinary 1-way ANOVA					
	Tukey's multiple comparisons test	Mean Diff.	95.00% CI of diff.	Significant?	Summary	Adjusted P Value
	SD vs. DIO	-0.9231	-1.396 to -0.4498	Yes	***	0.0002
	SD vs. DIO-STZ	-0.9957	-1.493 to -0.4986	Yes	***	0.0001
	DIO vs. DIO-STZ	-0.07254	-0.5281 to 0.383	No	ns	0.9159

Table S15. *Intraepidermal Nerve Fiber Density*

Group	Cage	Mouse ID	IENF (mm^2) 36wk
SD	AA	3268	49.01
	AA	3269	48.25
	AA	3270	x
	AA	3271	x
	AA	3272	51.13
	BB	3273	63.17
	BB	3274	55.09
	BB	3275	50.61
	BB	3276	55.54
	BB	3277	62.16
DIO	CC	3278	40.45
	CC	3279	40.70
	CC	3280	x
	DD	3281	40.44
	DD	3282	49.72
	DD	3283	43.60
	EE	3284	39.53
	EE	3285	34.43
	FF	3286	54.13
	FF	3287	42.05
DIO-STZ	GG	3288	35.02
	GG	3289	31.08
	GG	3290	38.64
	HH	3291	x
	HH	3292	37.32
	HH	3293	46.50
	II	3294	35.79
	II	3295	44.31
	JJ	3296	41.24
	JJ	3297	43.75

	Age (wk)	36wk
SD	Average (g)	54.4
	StDev	5.743
	n	8
	StError	2.031
	Max threshold	65.9
	Min threshold	42.9
DIO	Average (g)	42.8
	StDev	5.9
	n	9
	StError	1.950
	Max threshold	54.5
	Min threshold	31.1
DIO-STZ	Average (g)	39.3
	StDev	5.0
	n	9
	StError	1.7
	Max threshold	49.4
	Min threshold	29.2

Brown-Forsythe test	
F (DFn, DFd)	0.09764 (2, 23)
P value	0.9074
P value summary	ns
Are SDs significantly different (P < 0.05)?	No
→ Proceed with ordinary 1-way ANOVA	

Tukey's multiple comparisons test	Mean Diff.	95.00% CI of diff.	Significant?	Summary
SD vs. DIO	11.59	4.835 to 18.34	Yes	***
SD vs. DIO-STZ	15.07	8.324 to 21.83	Yes	****
DIO vs. DIO-STZ	3.488	-3.061 to 10.04	No	ns

Disease Models & Mechanisms • Supplementary information
